# Atypical Teratoid Rhabdoid Tumor: Two Case Reports and an Analysis of Adult Cases with Implications for Pathophysiology and Treatment

**DOI:** 10.3389/fneur.2017.00247

**Published:** 2017-06-20

**Authors:** Christopher Dardis, Jared Yeo, Kelly Milton, Lynn S. Ashby, Kris A. Smith, Shwetal Mehta, Emad Youssef, Jenny Eschbacher, Kathy Tucker, Laughlin Dawes, Neil Lambie, Elizabeth Algar, Elizabeth Hovey

**Affiliations:** ^1^Department of Neurology, Barrow Neurological Institute, Phoenix, AZ, Unites States; ^2^University of New South Wales, Sydney, NSW, Australia; ^3^Department of Neurosurgery, Barrow Neurological Institute, Phoenix, AZ, United States; ^4^Laboratory of Glial Tumor Biology, Barrow Neurological Institute, Phoenix, AZ, United States; ^5^Department of Radiation Oncology, Barrow Neurological Institute, Phoenix, AZ, United States; ^6^Department of Pathology, Barrow Neurological Institute, Phoenix, AZ, United States; ^7^Hereditary Cancer Clinic, Prince of Wales Hospital, Randwick, NSW, Australia; ^8^Department of Diagnostic Radiology, Prince of Wales Hospital, Randwick, NSW, Australia; ^9^Department of Anatomical Pathology, Prince of Wales Hospital, Randwick, NSW, Australia; ^10^Hudson Institute of Medical Research, Clayton, VIC, Australia; ^11^Department of Medical Oncology, Nelune Comprehensive Cancer Center, Prince of Wales Hospital, Randwick, NSW, Australia

**Keywords:** teratoma, rhabdoid tumor, adult, pregnancy, statistical data analysis, statistical data interpretation, etiology

## Abstract

We present the first quantitative analysis of atypical teratoid rhabdoid tumors (ATRT) in adults, including two patients from our own institutions. These are of interest as one occurred during pregnancy and one is a long-term survivor. Our review of pathological findings of 50 reported cases of adult ATRT leads us to propose a solely ectodermal origin for the tumor and that epithelial–mesenchymal transition (EMT) is a defining feature. Thus, the term ATRT may be misleading. Our review of clinical findings shows that ATRT tends to originate in mid-line structures adjacent to the CSF, leading to a high rate of leptomeningeal dissemination. Thus, we hypothesize that residual undifferentiated ectoderm in the circumventricular organs, particularly the pituitary and pineal glands, is the most common origin for these tumors. We note that if growth is not arrested soon after diagnosis, or after the first relapse/progression, death is almost universal. While typically rapidly fatal (as in our first case), long-term remission is possible (as in our second). Significant predictors of prognosis were the extent of resection and the use of chemotherapy. Glial differentiation (GFAP staining) was strongly associated with leptomeningeal metastases (chi-squared p = 0.02) and both predicted markedly worse outcomes. Clinical trials including adults are rare. ATRT is primarily a disease of infancy and radiotherapy is generally avoided in those aged less than 3 years old. Treatment options in adults differ from infants in that cranio-spinal irradiation is a viable adjunct to systemic chemotherapy in the adult population. Given the grave prognosis, this combined approach appears reasonable. As effective chemotherapy is likely to cause myelosuppression, we recommend that stem-cell rescue be available locally.

## 1 Introduction

### 1.1 History of ATRT

ATRT, a cancer of the CNS, was christened by Rorke et al. in 1996, following a review of 52 pediatric cases (1). We may trace the first appearance of the term “atypical teratoma” to four decades earlier, where it was recognized to occur in the pineal gland (2). The “atypical” refers descriptively to the “teratoid” part of the tumor. This appears to show elements of two germ cell layers (ectoderm, i.e., primitive neuroepithelial, epithelial, and mesoderm, i.e., “rhabdoid”) but is otherwise quite different from classical teratoid tumors. “Rhabdoid” (Greek, “rod-like”) refers to the similarity, on microscopy, to tumors showing skeletal muscle differentiation.

The biology of and treatment strategies for ATRT (focusing on children) have been the subject of a recent, thorough review. Although the prognosis in children has traditionally been dismal, with few long-term survivors, developments over the last 10 years have, at least for children, been encouraging (3). Recently, mutations in the chromatic remodeling complex *SMARCB1* (and, as a rare alternative, of *SMARCA4*) have been identified in the great majority of cases of ATRT (4). Such mutations appear necessary for the development of ATRT; no other consistent genetic abnormalities have been identified.

### 1.2 ATRT in Adults

While ATRT is the most common malignant CNS tumor in children aged <1, cases in adults (i.e., age >18) are rare—we estimate <1/1,000,000 lifetime risk (5). As such, these patients are presented in detail as case reports or in small case series. There are no molecular markers recognized as unique to adults rather than children; in both cases, loss of INI1 staining via immunohistochemistry (IHC) is considered sufficient for diagnosis in the appropriate context.

### 1.3 Rhabdoid Tumors

ATRT is often described as analogous to “malignant rhabdoid tumor of the kidney,” a tumor recognized since at least the early 1980s (6). Other CNS tumors with rhabdoid histology include the “epithelioid glioblastoma with rhabdoid component” as well as the “rhabdoid” variants of more common tumors: meningioma, carcinoma, chordoma, and even sarcoma (7).

### 1.4 EMT

Epithelial–mesenchymal transition (EMT) was initially identified as a phenomenon in developmental biology. The occurrence of EMT in certain cancers has been suggested since 1978, although it only began to receive widespread attention 10 years later (8). The role of EMT in carcinogenesis has been the subject of an extensive review (9). EMT appears to be crucial to the development and maintenance of a pool of slowly dividing, chemoresistant, “cancer stem cells.” We hypothesize that the “rhabdoid” appearance of the tumors above reflects EMT.

## 2 Case Reports

### 2.1 A Case in Pregnancy

#### 2.1.1 Presentation

Our first patient was a 29-year-old woman who developed hoarseness during week 20 of her first pregnancy, which deteriorated over the subsequent 5 weeks. Over the course of 1 day, she developed weakness of the right arm and leg. A CT performed elsewhere showed the presence of an extra-axial left cerebello-pontine mass. This was interpreted as a probable meningioma, so further treatment was deferred.

**Figure 1:**
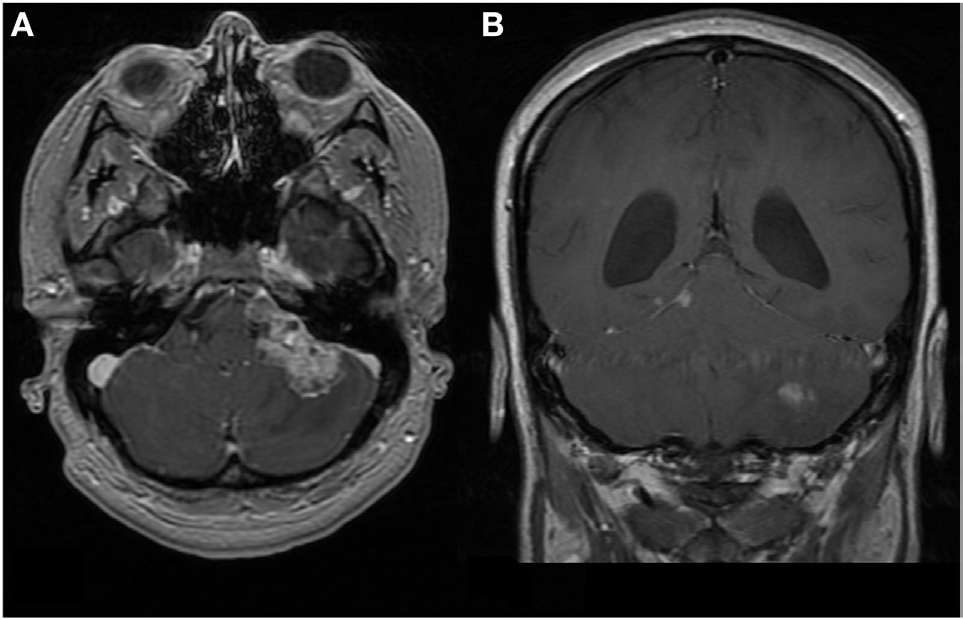
First patient. MRI brain at time of diagnosis, 1.5 T. Images are T1-weighted and Gd-enhanced. **(A)** Axial: heterogeneous extra-axial enhancement is seen in the left cerebello-pontine angle, with some local compression. **(B)** Coronal: enhancement is seen along the tentorium cerebelli on the right, which is characteristic of leptomeningeal spread. The left cerebellar lesion appears intraparenchymal.

**Figure 2:**
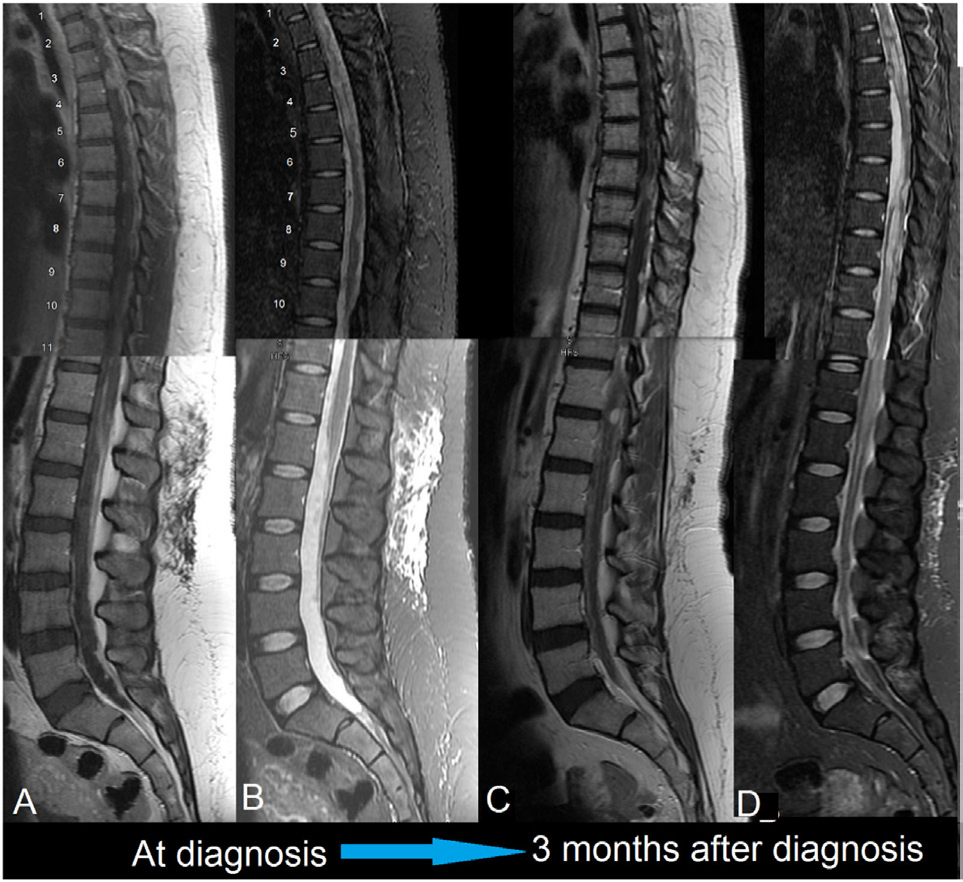
First patient. MRIs of spine, 1.5 T, sagittal. **(A)** Gd-enhanced: diffuse, nodular enhancement is seen throughout. This is striking dorsally and at the conus medullaris. **(B)** T2-weighted: the thoracic cord is swollen with intra-medullary edema. This appears to be a reaction to the surrounding leptomeningeal tumor; there may also be an element of cord compression in the thoracic cord. **(C)** Gd-enhanced: disease in the upper thoracic cord is much improved following radiotherapy. Progression has occurred around the untreated cord caudally. **(D)** T2-weighted: similarly, intra-medullary edema has improved in the treated areas but worsened elsewhere.

At 35 weeks gestation, she developed status migrainosus and worsening right leg weakness. Her voice had become weaker and she coughed intermittently when swallowing. Paralysis of her left vocal cord was demonstrated via laryngoscopy. Her MRIs at this time are shown in Figures 1 and 2. A cesarean section was performed in anticipation of neurosurgery and a healthy baby boy was delivered.

#### 2.1.2 Initial Treatment

A left retro-sigmoid craniotomy was performed. Pathology, showing loss of immunohistochemical staining for INI1, confirmed ATRT. Her spinal MRI showed leptomeningeal metastases in the cervical and thoracic cord. She rapidly developed hydrocephalus; a shunt and Ommaya reservoir were placed 1 week after surgery.

Multimodal treatment was instituted. Systemic chemotherapy involved two cycles (of 14 days) of ICE (ifosfamide, carboplatin, etoposide). Following the second cycle, she developed pancytopenia and sepsis, and thereafter no further systemic treatment was pursued.

Intrathecal chemotherapy was with liposomal cytarabine 50 mg via Ommaya every 2 weeks; she received 6 doses in total. Intensity-modulated radiotherapy to the cerebellum was 54 Gy in 30 fractions; conformal radiotherapy to the cord at C5–T6 vertebral levels was 30 Gy in 10 fractions. Disease in the brain and upper spinal cord improved overall with treatment, except for one focus in the pre-pontine cistern which grew in a plaque-like fashion.

#### 2.1.3 Treatment at progression

In view of progressive weakness of the legs, she received an additional 30 Gy in 10 fractions to the spinal cord at T12–L4. Within a month of completing this treatment, she developed a flaccid paraplegia with loss of bladder and bowel control and a sensory level at T10. Her MRI confirmed progression (Figure 2). Thereafter, she was cared for by Hospice and died 5 months following her initial surgery.

### 2.2 A Case with a Durable Response to Treatment

#### 2.2.1 Presentation

**Figure 3:**
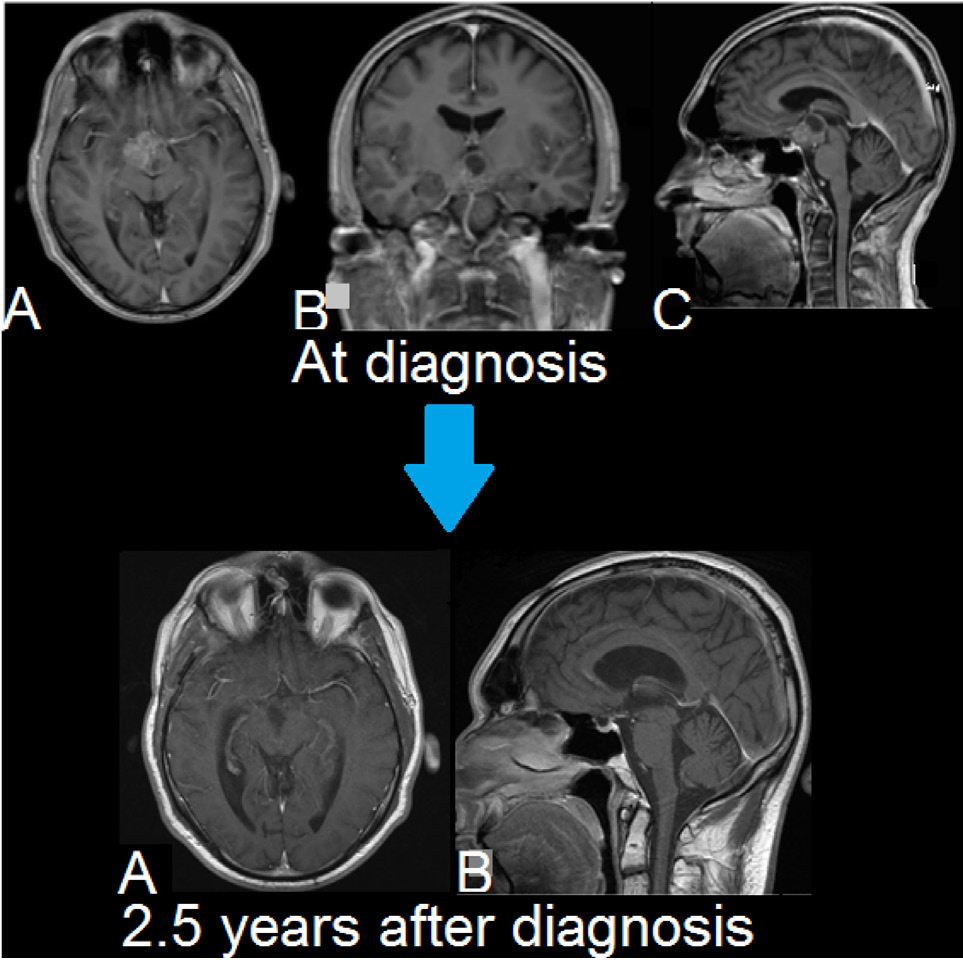
Second patient. MRIs of brain. Upper row **(A–C)**: T1-weighted, Gd-enhanced (magnetization-prepared rapid gradient-echo (MPRAGE)). A suprasellar and interpeduncular lesion is seen with mixed solid and cystic (lobulated) elements. There is heterogeneous enhancement of intermediate intensity. Lower row **(A,B)**: T1-weighted, Gd-enhanced. There is no evidence of tumor recurrence. Smooth linear enhancement is present at the resection site, which is consistent with the long-term changes seen following surgery.

Our second patient was a 35-year-old man who presented with blurred vision that had been present for 3 months. His MRI at this time, showing a suprasellar mass, is shown in Figure 3.

**Figure 4:**
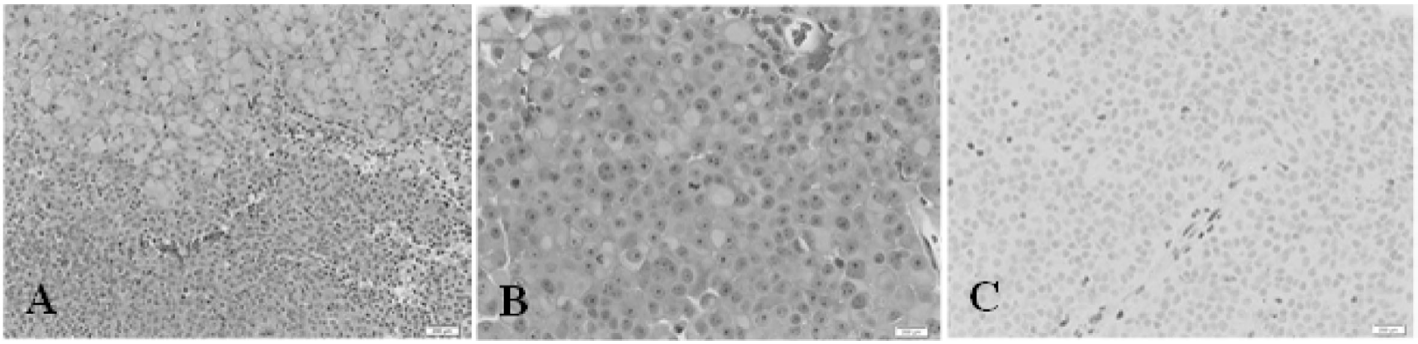
Second patient. Immunohistochemistry. **(A)** Hematoxylin and eosin (H&E) stain, 200× magnification. The tumor comprises two main elements: sheets of large cells, many with a rhabdoid appearance (top) and small, more primitive-appearing cells (bottom). **(B)** (H&E) stain, 400×. This shows large cells, many with rhabdoid features and central mitoses. **(C)** INI1 stain, 400×. There is no nuclear staining in the tumor cells. A positive control is provided by the endothelial cells in the vessel at the center, and in other scattered glial and inflammatory cells.

He underwent craniotomy, via a pterional approach, for resection of the tumor. The pathology is shown in Figure 4. This shows how ATRT may be suspected on the basis of hematoxylin and eosin (H&E) staining alone. The diagnosis was confirmed by IHC for INI1.

A homozygous mutation in *SMARCB1* was confirmed on Sanger sequencing. This was performed on the fresh-frozen, paraffin-embedded tissue. The sequencing covered the coding region and 5′ and 3′ splice sites from nucleotide 37 to 47,120 in the reference sequence NT_011520.gbk (10). The mutation was described as NM_003073.3: c.1148delC, NP_003064: p.P383fs*96. Occurring in exon 9, this causes a *SMARCB1* protein frame-shift and the insertion of additional amino acids at the 3′ end of the protein. This mutation is predicted to lead to inactivation of this protein in the tumor.

#### 2.2.2 Initial Treatment

MRIs performed following surgery demonstrated a nodule in the third ventricle that was believed to be persistent malignancy, as well as a nodule in the upper lumbar spinal cord, most likely a sign of leptomeningeal metastasis. Hence, cranio-spinal radiotherapy was given at a dose of 36 Gy in 20 fractions. Localized boosts were given to the primary site (20 Gy in 12 fractions) and the lumbar spine (8 Gy in 4 fractions). This treatment was completed 3 months following his initial surgery.

He proceeded to receive chemotherapy using the St. Judes ATRT protocol. This was developed for children aged >3 and comprises 4 cycles of cisplatin with high-dose cyclophosphamide and vincristine, with autologous peripheral blood stem-cell transplantation (11). This took 5 months to complete.

Since completing treatment there has been no sign of residual/recurrent disease at 2.5 years follow-up, clinically or on MRI, as seen in Figure 3.

Since his initial resection, he has suffered from panhypopituitarism, a left homonymous hemianopia and short-term memory impairment. He also developed a peripheral neuropathy following chemotherapy, most likely due to vincristine.

## 3 Methods

### 3.1 Review of Adult Cases

A literature search was performed using PubMed and Google Scholar to search for all reported cases of ATRT in adults. All 50 cases were tabulated; this is given as Supplementary Material (worksheet d1 in data sheet 1.xlsx). Many of these reports include similar tables; however, we noted a number of inconsistencies in these tables and ours is based on our reading of the original cases. While many prior authors have reported the ‘classical’ features of ATRT via IHC (see below), we are the first to tabulate *all* pathological variables for each case.

#### 3.1.1 Clinical Variables

In each case, we recorded age, gender, and location as well as presence of leptomeningeal metastases (LM) and details of treatment at initial diagnosis and time of first progression. As above, a meaningful response to treatment was rare if the disease progressed again after “second-line” approaches. ‘Third-line’ treatments of any kind were exceptional and were not recorded.

The diagnosis of LM was based on clinical details and/or imaging features, e.g., in the case report of Wang et al. recurrence was spinal (distal from the original tumor) and appears to have originated from the leptomeninges on MRI, with subsequent intra-medullary spread (12).

A number of nominal (categorical) variables were ‘collapsed’ as this was such a small data set. Location, for example, initially had 15 values; we also analyzed this as a 5- and 3-category variable in order to look for more general patterns. We also assessed whether the location was ‘central’ vs. clearly lateralized and whether the tumor was “next to CSF” based on imaging findings. Similarly, the extent of resection was considered as an ordinal variable (gross total > sub-total resection > biopsy) as well as binary (surgery or not). We analyzed age as binary (i.e., ≥40 years when diagnosed) as well as a continuous numeric variable.

#### 3.1.2 Pathological Variables

We consider here only the pathology at the time of diagnosis of ATRT. We note that only 2/40 (5%) of patients in our series had an autopsy performed (where we could infer this information). A summary table of all pathologic results is given as Supplementary Material (worksheet ihc1 in data sheet 1.xlsx). Most of these variables relate to the presence of certain antigens via IHC.

For IHC, a variety of antibodies and names were used to assess the presence of various elements. We have standardized this by recording the antigen being tested. Keratin staining, in particular, used a range of antibodies and was variously reported by antibody, by weight of the stained keratin, by keratin type or at times as “keratin present.”

### 3.2 Classification of IHC

#### 3.2.1 Diagnosis of ATRT

Loss of INI1 protein expression (via IHC) is considered a defining feature of ATRT. Partial deletion of chromosome 22 (detected via FISH), where the gene (*SMARCB1*) is located, is considered as an equivalent test. Both of these tests began to be used widely after the year 2000. While both tests are considered to have a specificity of 100% for ATRT (in the appropriate setting), a negative result does not exclude the diagnosis. Such testing was reported in 23/50 (46%) of the cases we reviewed.

Prior to the advent of INI1 testing, the diagnosis was suspected on the basis of the appearance on standard H&E staining. Staining for the “classical” antigens vimentin, epithelial membrane antigen (EMA), and smooth muscle actin (SMA) was typically used as confirmatory. Both vimentin and SMA are classically associated with *mesenchymal* cells, although this does not imply a *mesodermal* origin for such cells. For example, constitutive expression of vimentin is recognized in arguably the most-studied *glial* cell line, U-251 (13). Again, a negative test result for one of these antigens does not exclude the diagnosis.

#### 3.2.2 IHC and Differential Diagnosis

While the diagnosis of ATRT is often quickly suspected in an infant, the initial differential diagnosis in adults is typically far wider. Thus, the range of IHC stains employed in adult cases is far greater and more variable. In total, 81 tests (using IHC) were performed in our case series. In 19 (23%) cases, the antigen of interest was tested for in just one specimen.

IHC often included tests for metastasases, germ cell tumors, neuro-endocrine tumors, and other primary tumors of the nervous system (glial, medulloblastoma, neuroblastoma). Testing for a wider range of antigens (in adults vs. children) gives us a greater insight into the pathophysiology of ATRT.

Other possible differential diagnoses in adults may include primitive neuroectodermal tumor (PNET), choroid plexus carcinoma, rhabdoid meningioma, and germinoma. However, these entities generally have more characteristic immunohistochemical staining patterns with specific markers than the poly-phenotypic patterns seen in ATRT; also INI1 staining is retained.

#### 3.2.3 Differential: Rhabdoid Glioblastoma

Tumors resembling ATRT, staining with glial fibrillary acidic protein (GFAP) as well as vimentin, SMA and EMA have been suggested to represent rhabdoid glioblastoma (GB) rather than ATRT (7). EMT in the setting of GB appears plausible as an entity distinct from ATRT. GB with EMT *may* have been misdiagnosed as adult ATRT (prior to the advent of testing for INI1), although this appears unlikely given the clinical and histological findings in our cases.

#### 3.2.4 Germ Cell Layers

We used IHC to determine the putative germ cell layer of origin of each specimen. In so doing, we were guided by the tissues that are known to stain for an antigen under “normal” conditions. The website accompanying the textbook “Pathology Outlines” was particularly helpful in this regard (14).

Some stains are not restricted to one germ cell layer. An example is keratin 8, which predominantly occurs in ectodermal tissue but is also recognized in endodermal tissue (15).

#### 3.2.5 Classification

We thus classified pathological findings into the following groups:

1. ‘ATRT specific’
  - ‘Defining’ features (loss of INI1 or del(22))
  - ‘Classical’ features (vimentin, EMA, SMA)
2. Ectoderm
  - Neural crest (neuronal, glial, neuro-endocrine, melanoma markers)
  - Other ectodermal
3. Endoderm
4. Keratins
5. Mesoderm
  - Mesenchymal
  - Leukemia/lymphoma markers
6. Germ cell
7. Others (found in a variety of tissues).

### 3.3 Quantitative Analysis

Data analysis was performed using R (RRID:SCR_001905) (16–18). The complete analysis is available as Supplementary Material (data sheet 2.pdf). Nominal variables are given as fractions (percentage) and continuous variables as median (range).

We did not attempt to correct for multiple hypothesis testing, as the present work is exploratory (19). As the sample size is small, *a priori* we considered p values of <0.1 to be potentially worth reporting.

#### 3.3.1 Associations

We began by looking at the significance of *all* two-variable associations, using standard measures of strength and significance of association as follows:

**Table:** 

Variable 1	Variable 2	Correlation	p Value
Nominal	Nominal	Cramer’s V	Chi-squared
Numeric	Numeric	Pearson’s r^2^	t-Test
Nominal	Numeric	Pearson’s r^2^	F-Test

#### 3.3.2 Survival analysis

Time-to-event data were analyzed used the proportional-hazards model (PHM). We looked at the following outcomes:

1. time to progression
2. overall survival
3. time from progression to death (to assess the effects of second-line treatments).

Given the small number of observations and deaths, over-fitting was a problem with multivariate PHMs.

Survival times are reported as a median with 95% confidence intervals (CI). Effect size is given as hazard ratio (HR, relative to control) and the p value is from the score test (or Wald test for multivariate models).

#### 3.3.3 Missing Data

Given such a large quantity of missing data, we tried to compensate in a number of ways:

- Recursive partitioning ‘looks ahead’ at survival to find the best split for the given data. It is particularly well suited to data sets where there is near-perfect prediction of outcome by certain variables. It also allows for a clear ordering of variables in terms of predictive importance (20).
- “Multivariate imputation by chained equations” uses repeated regression on the existing variables to estimate missing values. The technique does have a tendency to strengthen existing associations rather than developing new insights (21).
- The “nested cohort” design allows for more ‘accurate’ estimates from a PHM where a covariate is observed for only some subjects (the cohort). Sampling is stratified by a variable that is available on all cohort members.
  This allows for frequency matching on confounders, or oversampling on the extremes of surrogate [markers] for exposures, to improve efficiency (22).
  That is, this technique allows one to control for major confounders in order to arrive at a more meaningful estimate of effect size and probability. In our case, we sought to better determine the effects of various treatments using a sampling scheme based on:
  1. the presence of LM or GFAP staining—as these were the major confounders of treatment
  2. the occurrence of death when the last recorded follow-up was performed—to try to control for censoring, i.e., the fact that certain patients were not followed up until death had occurred.

## 4 Results

### 4.1 Demographics

The 50 patients reviewed (including the present cases) had a median age of 32 (18–65); 27 (54%) were females. Of those of childbearing age, 3/20 (15%) were pregnant when the diagnosis was made.

### 4.2 IHC

Some of the more significant findings on IHC are given in Table 1. As above, the complete table, with explanations, is given in (data sheet 1.xlsx).

**Table 1:** Immunohistochemistry (IHC) of adult ATRT.

Stain	Long name	+ve	n	%
**‘Defining’ features of ATRT**				
del(22)(q)	22q11.2 distal deletion	13	15	87
INI1	Integrase interactor 1/SMARCB1/hSNF5	1	23	4
**‘Classical’ ATRT markers**				
vim	Vimentin	33	33	100
EMA	Epithelial membrane antigen	29	35	83
SMA	Smooth muscle actin	18	32	56
**Neuronal**				
S100	100% soluble in (NH4)2-SO_4_	13	27	48
nfp	Neurofilament protein	10	15	67
nse	Neuron-specific enolase	2	6	33
Nestin		2	2	100
**Glial**				
GFAP	Glial fibrillary acidic protein	12	30	40
**Neuro-endocrine**				
Synaptophysin	Major synaptic vesicle protein p38	6	22	27
**Keratin**				
ker	Keratin (any, including type not specified)	20	49	41
ck8	Keratin 8; 52 Da	12	16	75
**Mesenchymal**				
Desmin		0	19	0
Myoglobin		0	4	0
**Others**				
CD34	Hematopoietic progenitor cell antigen CD34	3	11	27
CD99	Single-chain type-1 glycoprotein	2	7	29
p53	Cellular tumor antigen p53	4	5	80

*+ve, positive result, i.e., staining for antigen present; n, number of cases tested; %, percentage positive/number tested*.

Defining features of ATRT were found in almost all cases where these were assessed: loss of INI1 in 23/24 (96%), del22q in 13/15 (87%). It is tempting to consider those cases where these were absent as “false negatives”; we will return to this in the discussion.

Of the “classical” features, only vimentin was universally positive (33/33) in our series. Germ cell markers, where assessed, were uniformly negative. Keratins were variably expressed; keratin 8 was found in the highest proportion of samples assessed, 12/16 (75%). As above, we cannot use this help attribute a germ cell layer of origin to a specimen (15).

#### 4.2.1 Nestin

Markers of neuronal, glial, and neuro-endocrine differentiation were relatively common (*c*. 25–75%). Of particular interest is the presence of nestin in 2/2 cases studied. This is considered a marker of ‘neuroepithelial stem cells,’ is active in embryogenesis, and is downregulated in maturity, when expression of neurofilament protein and GFAP occur.

Interestingly, nestin is also expressed in developing muscle (presomitic mesoderm), to be replaced by desmin in maturity. Yet, desmin was universally absent in our sample (0/19). Other markers of mature muscle differentiation were also lacking, as well as the myogenic regulatory factor MYOD1 (0/2 cases), which is involved in myogenesis.

#### 4.2.2 CD34

The hematopoietic system is thought to be derived from mesoderm; as such the presence of markers more typically used in the diagnosis of leukemia and lymphoma in our sample may at first appear anomalous. However, CD34 is recognized to occur in tumors of neuroepithelial origin, particularly those of childhood (23). It is also expressed on endothelial cells.

#### 4.2.3 CD99

CD99 has been less well studied in CNS tumors. It is recognized to occur in “primary neuroepithelial tumors of the kidney,” an entity we believe to be closely related to ATRT (24). It is also present in ependymoma, peripheral primitive neuroectodermal tumor, and in the rare chordoid glioma, all of which are of neuroepithelial origin (25–27).

### 4.3 Associations

A number of striking associations are given in Table 2. Additional relationships of significance statistically but of debatable importance biologically are given as in the Supplementary Material as data sheet 2.pdf.

**Table 2:** Significant (*p* < 0.1) two-variable associations.

					Chi-square p	

ASSOCIATIONS WITH LOCATION						

Gender	4th Ventricle	Lateral Ventricle	Pineal	Pituitary	Spinal Cord	
Male	4	13	2	0	3	0.046
Female	3	7	4	10	3	

**Surgery, radiation, and chemotherapy**						
No	3	14	0	6	4	0.007
Yes	3	3	5	4	0	

**Gender**	**Lateral**	**Mid-line**				

Male	15	7				0.043
Female	9	17				

**Age**	**Brainstem**	**Lateral ventricle**	**Spinal cord**			

18–40	12	22	1			0.004
>40	6	4	5			

**Leptomeningeal metastases at recurrence**						
No	2	16	2			0.039
Yes	6	5	1			

**OTHER ASSOCIATIONS**						

	**Leptomeningeal metastases**					
**GFAP**	**No**	**Yes**				

−ve	16	2				0.018
+ve	5	7				

	**Synaptophysin**					
**SMA**	**−ve**	**+ve**				

−ve	9	0				0.031
+ve	5	6				

	**Neurofilament protein**					
**Gender**	**−ve**	**+ve**				
Male	5	4				0.094
Female	0	6				

*GFAP, glial fibrillary acidic protein; SMA, smooth muscle actin*.

#### 4.3.1 Demographics and Location

Where the tumor was in or next to the pituitary, 10/11 cases were in females (91%). The predilection of adult ATRT for this location has been recognized, although the sex-specificity seen here is novel (28).

Tumors of the spinal cord were associated with increasing age (Pearson’s r = 0.43, F test p = 0.002).

#### 4.3.2 IHC

Tumors with LM when diagnosed were much more likely to show GFAP staining (n = 30, chi-squared p = 0.02), implying that glial differentiation is a major risk factor for this complication.

All tumors that showed synaptophysin staining also stained for SMA (n = 20, chi-squared, p = 0.03). Synaptophysin was the only neuroendocrine marker commonly assessed in the sample; this finding suggests that differentiation along such lines is associated with a greater tendency to manifest a mesenchymal phenotype.

Findings regarding neural differentiation were difficult to interpret. Neurofilament protein (NFP) staining was present in all female patients (6/6) vs. 4/9 males (chi-square p < 0.1). Strangely, staining for NFP and neurospecific enolase (NSE) appeared mutually exclusive, i.e., where one was positive the other was negative, although there were only 4 subjects where both were checked. No cases where NFP staining was absent stained for GFAP (n = 15).

### 4.4 Treatment

#### 4.4.1 Surgery

Surgery (SX) at diagnosis was a cornerstone of treatment; in only one case was this not undertaken, where imaging initially suggested vestibular schwannoma and radiotherapy was used instead (12). Gross total resection (GTR) was reported in 10/43 (23%) of cases. This was most common for those tumors located next to a lateral ventricle. Subtotal resection was most common for tumors of the pituitary and pineal gland.

#### 4.4.2 Adjuvant Treatment

Radiotherapy (RT) was undertaken in 33/42 (79%) and systemic chemotherapy (CT) in 17/42 (40%). Cranio-spinal irradiation (CSI) was performed in 5/34 (15%). All three approaches (SX, RT, and CT) were used in 15/42 cases (36%). This multimodality approach was most commonly used for those tumors next to a lateral ventricle (Table 2).

Intrathecal CT was used in just two cases other than our first patient. In contrast to our use of liposomal cytarabine, these two patients were treated with small quantities of methotrexate: three and one dose(s), respectively (29, 30).

#### 4.4.3 Treatment at recurrence

When disease re-occurred/progressed, lower proportions of patients received treatment. Additional SX was attempted in 12/26 (46%), RT in 13/26 (50%) and CT in 11/26 (42%).

### 4.5 Survival

The median time to progression (TTP) was 5 months (95% CI 3–18). Overall survival (OS) was 23 months (14–56). Time from progression until last observation or death (time from progression to death, TPD), in the 27 patients where this was applicable and available, was 8 months (5–28). The fractions of patients without progression, and surviving, at various points in time from diagnosis are shown in Table 3.

**Table 3:** Percentages of patients without progression, and surviving, at timepoints following diagnosis.

Time from diagnosis	Progression-free (%)	Surviving (%)
6 months	46	76
12 months	33	64
2 years	20	39

#### 4.5.1 Long-term Survivors

Apart from our second patient, two others are reported to have survived for more than 3 years (31, 32). Unlike our patient, both re-occurred on multiple occasions following their initial treatment. The first was a frontal lesion that required complete excision 6 times (and once in the contralateral frontal lobe) as well as receiving radiotherapy, Gamma Knife radiosurgery and systemic chemotherapy; after 7 years of relapsing disease, she remained disease free for another 10 years. The other was a parietal lesion which re-occurred 3 times over the course of 9 years and which appeared stable 1 year after most recent treatment. Interestingly, this patients’ tumor was diagnosed and treated as glioma, at presentation and at first re-occurrence. The diagnosis of ATRT was subsequently made in retrospect due to the absence of INI1 by IHC in all specimens.

#### 4.5.2 Clinical Variables and IHC

**Figure 5:**
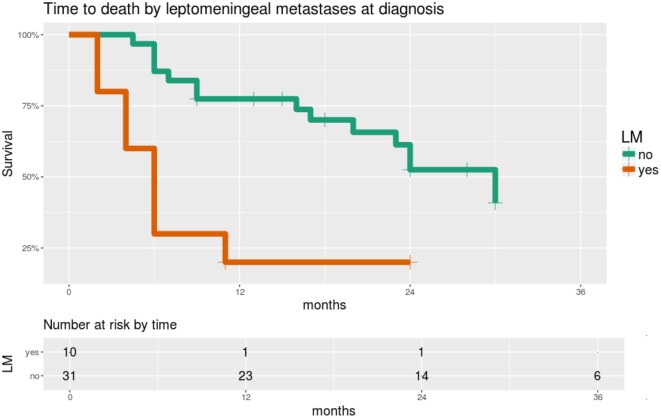
Overall survival is significantly worse in patients with leptomeningeal metastases (LM) at the time of diagnosis. Time scale is shown in months. Hazard ratio 4.4, score test p = 0.0005.

Significant predictors of survival in PHMs are shown in Table 4. Of note, LM and GFAP staining appear as significant predictors in almost all of these models. While LM and GFAP are correlated, LM appears to be the more important predictor, particularly in multivariable PHMs. The effect of LM on OS is shown in Figure 5.

**Table 4:** Leptomeningeal metastases and GFAP staining are significant predictors in most proportional-hazards models.

	Variable	n	HR	p
**Uni-variable**				**Score**

TTP	RT	33	0.13	<0.01
	SX	33	Ordinal	0.02
	GFAP	22	1.9	0.19
	LM	33	2.4	0.04
	GTR	33	0.86	0.75
OS	LM	41	4.6	<0.01
	GFAP	27	3.1	0.04
	SX	40	Ordinal	<0.01
	SRC	40	0.25	0.02
	Pregnant	18	4.5	0.05
	GTR	39	0.55	0.3
TPL	LM	27	6.7	<0.01
	GFAP	18	7.5	<0.01

**Multivariable**				**Wald**

TTP	LM		4.4	0.01
n = 21, e = 14	RT	21	0.32	0.13
	GTR		0.41	0.20
	GFAP		2.3	0.18
OS	LM	27	5.5	0.02
n = 27, e = 15	GFAP		2.4	0.25
	GTR		0.27	0.07
	CT		0.24	0.03
	RT		1.5	0.6
TPL	LM	18	3.7	0.4
n = 18, e = 14	GFAP		2.4	0.6

*p values: for uni-variable models = score test; for multivariate models = Wald test*.

*HR, hazard ratio; TTP, time to progression; OS, overall survival; TPL, time from progression to last follow-up/death; n, number of observations; e, number of events (progression or death); RT, radiotherapy; SX, surgery (ordinal; HRs not shown); GFAP, glial fibrillary acidic protein; LM, leptomeningeal metastases; GTR, gross total resection (vs. other value for surgery); SRC, surgery, radiation, and chemotherapy; CT, chemotherapy*.

#### 4.5.3 Treatments

“Over-fitting” was a problem when using surgery as an ordinal-scale variable in multivariate models. Instead, we give this as “gross total resection?” (GTR, yes/no). Regarding OS, only SX and multimodality treatment (SX, RT, and CT, HR = 0.3) were significant predictors. Both GTR and CT were significant predictors in the multivariable PMH for OS.

In assessing the effects of treatment, we acknowledge that we are not “comparing like with like,” i.e., patients for whom a certain treatment is not possible will be expected to have a worse outcome. Regarding surgery, GTR is generally the goal, where possible; those for whom this is not feasible will tend to have a worse prognosis, no matter the approach to surgery. Similarly, those for whom CT is impossible will tend to have poorer survival outcomes. However, significant comorbidities or disabilities due to disease (thus limiting the use of CT) were exceptional at the time when CT was commenced.

### 4.6 Recursive Partitioning

**Figure 6:**
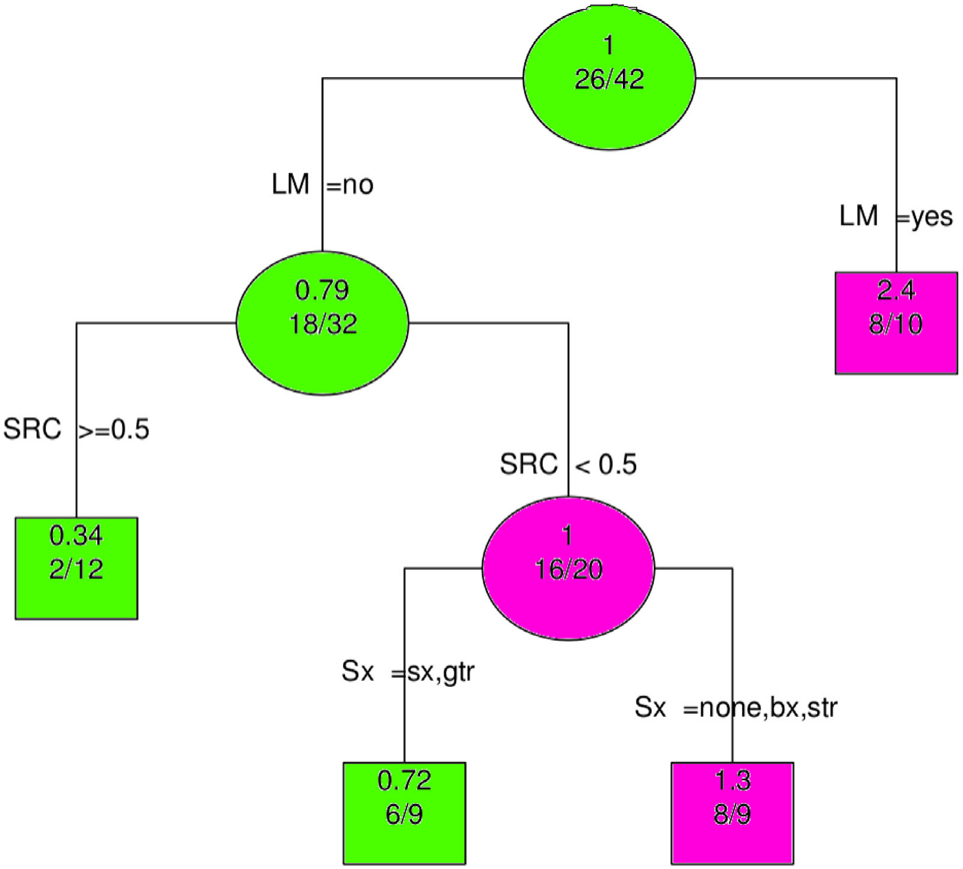
Recursive partitioning analysis shows LM to be the most important predictor of overall survival. Abbreviations: LM, leptomeningeal metastases; SRC, surgery, radiation and chemotherapy (≥0.5 means all modalities employed); Sx, Surgery; gtr, gross total resection; sx, surgery (extent unspecified); str, subtotal resection; bx, biopsy; none, no surgery. Key: circles, branching nodes; squares, terminal nodes; green, better outcomes; pink, worse outcomes; upper, no. predicted event rate; lower, no. no. events/no. at risk.

The most important predictor of OS was clearly LM; this is shown in Figure 6. When LM was excluded from the analysis, multimodality therapy (SX, RT, and CT) became the most significant variable (Supplementary Material, data sheet 2.pdf).

### 4.7 Multiple Imputation and “Nested” PHM

Applying these techniques to the potentially important predictors already identified tended to confirm the results above (data sheet 2.pdf).

The effects of the most common initial treatments on OS, when controlling for LM and GFAP (and death, in both cases), are shown in Table 5.

**Table 5:** Chemotherapy is the most important predictor of overall survival, when controlling for leptomeningeal metastases and GFAP staining (using ‘nested cohort’ proportional hazards models).

	Variable	HR	p
LM	CT	0.45	0.09
n = 39, e = 24	RT	0.56	0.3
	GTR	0.43	0.3
GFAP	CT	0.31	<0.01
n = 27, e = 15	RT	0.64	0.5
	GTR	0.31	0.1

*HR, hazard ratio; GFAP, glial fibrillary acidic protein; LM, leptomeningeal metastases; n, number of observations; e, number of events (deaths); CT, chemotherapy; RT, radiotherapy; GTR, gross total resection (vs. other value for surgery)*.

## 5 Discussion

### 5.1 Pathogenesis

#### 5.1.1 Location at Diagnosis and Implications for Anatomical Origin

ATRT in adults has a predilection for mid-line structures, particularly the pineal and pituitary glands. These are circumventricular organs, which are now recognized as a source of neural stem cells in adults (33).

ATRT may be said to be the prototypical tumor of infancy, in having the earliest onset and highest perinatal incidence of such tumors. Tumors of infancy in general are thought to arise from cells that have not completed the process of terminal differentiation and continue to undergo hyperplasia from the latter half of pregnancy to age 3 or so (34).

Our case series suggests that there remains a pool of slowly dividing ‘ectodermal stem cells’ in at least some of these circumventricular organs that remains capable of acquiring carcinogenic mutations throughout most of adulthood (35). The proximity of these organs to CSF may explain the close association of these tumors with such locations and their tendency to develop LM. Of note, when ATRT occurs in a cerebral lobe, it typically appears in communication with a lateral ventricle.

We suspect that in many cases the tumor has already spread via CSF prior to diagnosis. This would help to explain the proposal of such entities as “primary diffuse cerebral leptomeningeal ATRT” and “ATRT arising from the acoustic nerve”; these structures appear unlikely to be points of origin for ATRT (12, 36).

The strong association between pituitary involvement and female gender is consistent with the greater mitotic activity of the pituitary in females throughout the lifespan (37). We acknowledge there remains much work to be done in this area.

#### 5.1.2 IHC and Implications for Cell Type of Origin

As summarized in Table 1, findings on IHC suggest a tumor of ectoderm origin with EMT. For example, the combination of EMA (epithelial, 83%), vimentin (mesenchymal, 100%), and complete absence of markers of mature mesenchyme (desmin, myoglobin, 0%) are characteristic of such a phenotype.

In this regard, the name “ATRT” is somewhat misleading in that it is clearly not a teratoma and does not contain elements originating from mesoderm or showing skeletal muscle differentiation.

Classification of IHC by cell type and germ layer in adult ATRT has also been undertaken by Raisanen et al. (38). Their table is similar to our own, albeit with a smaller sample size. The authors do not attempt to infer any implications for pathogenesis from their table.

#### 5.1.3 Germline Mutations and del22q

Loss of INI1 is not sufficient to cause ATRT. This is shown by the lack of complete penetrance of ATRT in germline mutations (distal 22q11 microdeletion syndromes) (39). None of the cases of ATRT in this series, including our own, had germline analysis performed to look for a mutations or deletion of chromosome 22q. The only case we reviewed where this seemed likely was our case with a prolonged response to treatment. The patent had macrocephaly and a history of spinal schwannoma as a teenager. None of the other reported a personal or family history of dysmorphism or of tumors seen in the rhabdoid-predisposition syndrome.

Nonetheless, it has been recognized that germline mutations in *SMARCB1* germline mutations are not always inherited and unaffected adult carriers are recognized (40). In this series of 100 patients, those with germline mutations tended to present at a much younger age, at a median of 5 months (range birth—5 years) vs. 18 months (birth—17 years) for those with acquired mutations. However, in 4 of 7 cases of inherited mutations, the parents appeared completely unaffected. The authors also recognize germline/gonadal mosaicism in their sample, i.e., the mutation must have been present within parental gametes but not somatic cells.

Such germline mutations result in a phenotype known as “rhabdoid predisposition syndrome.” In addition to ATRT, carriers appear predisposed to rhabdoid tumors (renal and extra-renal), PNET, medulloblastoma, choroid plexus carcinoma and schwannoma (41). These authors also suggest the mutation as a cause for cases of Li–Fraumeni-like syndrome (patients with a childhood cancer or “sarcoma” aged <45 and a family history of cancer at a young age, in those lacking germline *TP53* mutations).

Thus, we recommend somatic analysis in addition to analysis of the tumor, even in adult patients (42). Analysis of parental somatic and germline DNA also appears desirable.

#### 5.1.4 INI1, del22q, and Epigenetic Subgroups

The only consistent genetic change in these tumors appears to be loss of INI1 due to *SMARCB1* mutations (which are variable) (4, 43). Mutation of *SMARCA4* is recognized as an alternative inciting event in rare cases, perhaps accounting for the single patient in our series with preserved INI1 expression. The lack of other consistent candidate oncogenic mutations has led to the recognition that epigenetic changes are crucial to pathogenesis. Three such epigenetic subgroups are now recognized (44). There were no adults in this recent work characterizing subgroups (age range: birth to 9.5 years). The subgroups are named based on the pathways most commonly upregulated, here shown with some of the genes whose expression is characteristically increased:

**Table:** 

ATRT-TYR	Melanogenesis pathway	*EZH2*, DNA methyltransferases (*DNMT*s), *CCND1, VEGFA, ERBB2*
ATRT-SHH	Hedgehog pathway	*EZH2, DNMT*s, *CDK6*
ATRT-MYC	*MYC* pathway	*MYC, HOX* genes, *EZH2, DNMT*s, *ERBB2*

Phenotypically, the ATRT-TYR subgroup is strongly associated with a supratentional location vs. ATRT-MYC; ATRT-SHH appears in both locations.

Further characterization of phenotype by subgroup may be facilitated by preserving the methylation status of DNA in the tumor specimen. We suggest that fresh-frozen material be obtained for this purpose where practical. Technical advances continue to make methylation profiling more practical in formalin fixed, paraffin-embedded samples.

#### 5.1.5 Chromatin Remodeling and EMT

EMT is known to result in a more ‘open’ chromatic structure (9). This is necessary to facilitate de-differentiation, i.e., the transcription of mRNA, which is typically unavailable to the differentiated epithelial cell. This includes a shift in energy (ATP) production from oxidative phosphorylation to glycolysis. This is necessary for the supply of energy of biosynthetic precursors, of a balanced redox status and appears necessary to maintain the undifferentiated state.

Chromatin remodeling complexes such as INI1 have been implicated in various cancers (45). INI1 is now recognized as a tumor suppressor, due to its mutation in tumors including choroid plexus carcinoma, medulloblastoma, primitive neuroectodermal tumor, and chronic myeloid leukemia. INI1 is a member of the SWI2/SNF2 remodeling complex, which is ATP dependent.

Loss of function of these chromatin remodeling complexes enables the development of the ‘open’ structure required for EMT. It remains unclear why loss of INI1 leads to diverging epigenetic phenotypes.

### 5.2 Clinical Trials

Ongoing and recently completed trials for this condition are tabulated in the recent review by Fruhwald et al. (3). The dearth of clinical trials enrolling adults >21 years old, just seven at the time of writing, is shown in Table 6.

**Table 6:** Ongoing clinical trials enrolling patients aged >21.

NCT ID	Agent	MOA	CA	MC	Age	Phase	n	Cohorts	
http://clinicaltrials.gov/show/NCT02601950	Tazemetostat	EZH2 I	No	Yes	>16	2	150	rhabdoid:	ATRT MRT RTK
								Refractory synovial sarcoma + SS18-SSX rearrangement	
								INI1 −ve:	EMPNST EMC Myoepithelial Ca Chordoma
								RMC Epithelial sarcoma	

http://clinicaltrials.gov/show/NCT01505569	1–3 cycles: Ifosfamide Etoposide →Carboplatin →Thiotepa	Autologous peripheral blood stem cell transplant	Yes	No	<70	SOC	20	r/r solid tumor	
								CNS tumor:	ATRT PNET age <3 High risk MB age <3 MB age <8 months Anaplastic MB
								Germ cell tumor	

http://clinicaltrials.gov/show/NCT00445965	^131^I-3F8 Ab	Ganglioside GD2	No	No	Any	2	131	CNS/LM tumor GD2 +ve	

http://clinicaltrials.gov/show/NCT00983398	Intra-arterial:		Yes	No	1–30	1/2	55	r/r CNS embryonal	ATRT PNET
	Melphalan Carboplatin Mannitol Thiosulfate	Alkylating DNA repair Osmotic Adsorbent						tumor:	MB Medulloepithelioma Pineoblastoma Ependymoblastoma
								Germ cell tumor	

http://clinicaltrials.gov/show/NCT02114229	Alisertib	Aurora A I	No	Yes	<22	2	180	ATRT (with chemoTx)	
								r/r:	ATRT MRT

http://clinicaltrials.gov/show/NCT02684071	Intraventricular methotrexate with systemic:		Yes	No	<22	2	10	r/r:	ATRT PNET
	Topotecan Cyclophosphamide	Topoisomerase I Alkylating							MB Ependymoma

http://clinicaltrials.gov/show/NCT02458339	Methotrexate via 4th ventricle	Anti-folate	Yes	No	1–21	1/2	18	r/r:	ATRT PNET MB Ependymoma CPCa

*NCT ID, National clinical trials identifier; CA, commercially available; MOA, mode of action; MC, multi-center (i.e., ≥3 sites); n, number of patients (estimated accrual); Ab, antibody; I, inhibitor; EZH2, enhancer of zeste homolog 2 (enzyme); SOC, standard of care/protocol; r/r, recurrent/refractory (progressive); MRT, malignant rhabdoid tumor; RTK, rhabdoid tumor of the kidney; EMPNST, epithelioid malignant peripheral nerve sheath tumor; EMC, extra-skeletal myxoid chondrosarcoma; RMC, renal medullary carcinoma; PNET, primitive neuroectodermal tumor; MB, medulloblastoma; CPCa, choroid plexus carcinoma*.

As a rare condition, even in children, there is some justification for ‘lumping’ ATRT with similar tumors. This is particularly rational when the intervention is relatively non-specific, e.g., radiotherapy, traditional ‘cytotoxic’ chemotherapy (and soon, perhaps, immunotherapy).

In the case of more ‘targeted’ treatments, the pool of analogous diseases will be much smaller. Two of the seven trials may be said to be target aspects of the biology of ATRT. It is striking that the trial of alisertib aims to inhibit aurora kinase A, an enzyme that has been implicated in EMT. This trial includes malignant rhabdoid tumors (MRT), which show EMT but do not lose INI1 protein (46). MRTs, as well as rhabdoid tumors of the kidney (also showing EMT), are included in the trial of tamezostat (an EZH2 inhibitor). While beyond the scope of the current work, we suggest that the term “rhabdoid” is synonymous with EMT. Interestingly, the trial of tamezostat considers other tumors lacking INI1 to be sufficiently similar to be worth including.

The other five trials that include ATRT are relatively ‘non-specific’ and compensate for its low incidence by combining it with other ‘serious’ tumors, typically also of childhood. For example, four of five include PNET and medulloblastoma.

### 5.3 Treatment

As ATRT is primarily a disease of infancy, co-ordination of treatment between pediatric and adult oncology services appears appropriate.

#### 5.3.1 Chemotherapy

ATRT usually occurs before CSI can be given safely, i.e., in those aged <3. Thus, CT has traditionally been a cornerstone of treatment. We suggest that a primary guiding factor be penetration into CSF (given the high prevalence of LM) as well as into the intraparenchymal tumor. As these tumors tend to disrupt the blood–brain barrier (BBB) and are often next to parts of the brain with no BBB, penetration of CT into normal brain parenchyma does not appear to be of prime concern.

In the rare cases when ATRT is localized and can be completely excised, local RT appears appropriate; arguably, a protocol similar to that used for glioblastoma can be adopted in such cases.

The most common approach to CT typically involves cytotoxic and relatively non-specific agents. Ifosfamide, carboplatin, and etoposide (ICE) was the most common regimen in our series, a protocol typically used for sarcoma and lymphoma. These agents have reasonable brain and CSF penetration (47–49). However, given the grave prognosis of this condition, a more aggressive approach appears warranted in adults, as in our second case.

#### 5.3.2 Radiotherapy

Several reports demonstrated the beneficial role of adjuvant RT after surgical resection in patients with ATRT (11, 50, 51). However, RT is a significant challenge in those younger than 3 years old (52).

The benefit of combining RT with SX in ATRT has been shown by Lau et al. (53). In a sample of 171 pediatric and 3 adult cases, they report an increase in median survival from 1.9 ± 0.4 to 5.9 ± 0.7 years in those who had RT in addition to SX. Buscariollo et al. confirmed the value of RT in another retrospective series, this with 144 patients, where median survival improved from 6 to 34 months (p < 0.001) (54).

Once a decision on the use of RT has been made for a patient with ATRT, factors such as timing, dosing, and technique need to be considered.

Chen et al. reported improved disease-free survival in patients who had a total dose more than 50 Gy (55). Significant improvement in median survival was reported in patients who had a shorter interval between SX and RT. RT delivered early vs. latein the course of treatment has also been associated with improved survival (11).

Proton therapy may be especially attractive in younger patients, in order to minimize the risk of late complications. Encouraging results with local conformal proton therapy have been reported by Bernstein et al. (56). Nine of ten patients with ATRT (with a median age of 1.8 years) had no evidence of disease, with a median follow-up of 27.3 months.

As distal relapse is associated with higher mortality and as the majority of distant relapses occur within the CNS, CSI is recommended is those older than 3 years old: 23.4 Gy to the neuraxis (with 54 Gy to the tumor bed) is recommended inpatients older than 3 years old. Younger patients have been treated using either no adjuvant RT or local-only RT, with a trend toward improved survival with the addition of RT.

#### 5.3.3 A Combined Approach

Promising results have been already been shown in children >3 with PNET undergoing CSI followed by high-dose CT and in the setting of recurrent CNS tumors (57, 58). Stem-cell rescue was required with these more aggressive regiments, either from peripheral blood or bone marrow. Given its known role as a radiosensitizer and excellent brain and CSF penetration, the use of temozolomide during radiotherapy also appears reasonable (59). Early use of intrathecal chemotherapy also appears rational. As it is well tolerated in long-term use, it may also be an acceptable approach in those patients who would not tolerate more aggressive forms of CT.

## 6 Conclusion

ATRT in adults carries a grave prognosis, particularly in those cases with leptomeningeal spread. We postulate that ATRT is a tumor of neuroectodermal origin that demonstrates mesenchymal transition.

Our cases indicate the possibility of improved outcome with more aggressive therapy. CSI should always be considered in adults. The use of stem-cell rescue allows for the use of more aggressive chemotherapeutic regimens than has hitherto been the case.

## Consent

Written, informed consent was obtained from both patients specifically for the publication of these case reports.

## Author Contributions

CD drafted the manuscript and performed the statistical analysis. CD and KM wrote the first case report and performed the literature review. JY and EH wrote the second case report. EA performed the Sanger sequencing. All the authors contributed to data interpretation and to the final draft of the manuscript.

## Conflict of Interest Statement

The authors declare that the research was conducted in the absence of any commercial or financial relationships that could be construed as a potential conflict of interest.

## Supplementary Material

Additional information is provided in supplemental files:

- data sheet 1.xlsx A spreadsheet with the following worksheets:
  - d1 Data set used for analysis.
  - k1 Key to data set.
  - ihc Immunohistochemistry.
- data sheet 2.pdf Statistical analysis.

Case reports and case series reviewed, but not specifically cited above (60–89).
