# Supplementary material for: Atypical Teratoid Rhabdoid Tumor: Two Case Reports and an Analysis of Adult Cases with Implications for Pathophysiology and Treatment
Source: Front Neurol. 2017 Jun 20;8:247. doi: 10.3389/fneur.2017.00247 (PMC5476998; doi:10.3389/fneur.2017.00247)
Supplement: Supplementary file 2 [file Data_Sheet_2.PDF]

Statistics for ATRT in adults

Chris Dardis

Wednesday 15th February, 2017

Contents

1 Key 2
2 Data 6
3 Useful functions 7
4 Missing data 9
5 Descriptives 10
6 Outcomes 19
7 Treatment 21
8 Exploratory analysis; binary associations 24
9 Striking associations 30
10 Time to progression (exploratory) 34
11 Time to progression 35
12 Overall survival (exploratory) 39
13 Overall survival 40
14 Progression to death (exploratory) 45
15 Progression to death 46
16 Recursive partitioning 49
17 Multiple imputation 51
18 Using Nested cohort to contol 53
19 Session Info 54

1 Key

```
suppressPackageStartupMessages(library("data.table"))
suppressPackageStartupMessages(library("xtable"))
options("xtable.booktabs"=TRUE)
nrow(k1 <- data.table(read.csv("k1.csv", stringsAsFactors=FALSE)))
```

[1] 182

```
options("xtable.booktabs"=TRUE)
options("xtable.table.placement"="H")
options("xtable.include.rownames"=FALSE)
options("xtable.NA.string"="NA")
k1 <- data.table(read.csv("k1.csv", stringsAsFactors=FALSE))
print(xtable(k1,
             align=c("l", "c", "l", "r", "l"),
             caption="Key to data",
             label="tab:key"),
      tabular.environment="longtable",
      floating=FALSE)
```

Short.name	Full.name	Values	Values...details
first	first author		
yr	publication year of case		
age	age at diagnosis		years
fert	fertile?	0	no
	i.e. female and aged 18-50	1	yes
preg	pregnant?	0	no
		1	yes
		NaN	not a number
			i.e. pregnancy impossible
lat	laterality	NA	not available (unknown)
		l	left
		c	central
		r	right
loc	location	sc	spinal cord
		t	temporal
		f	frontal
		cd	caudate
		pa	parietal
		t	temporal
		pi	pineal
		ss	suprasellar
		cb	cerebellum
		th	thalamus
		s	sella
		o	occipital
		pf	posterior fossa
		fp	fronto-parietal
		ft	fronto-temporal
		pt	parieto-temporal

		pit	pituitary
		cpa	cerebello-pontine angle
csf	tumor next to CSF?	0	no
		1	yes
		NA	not available
	DISEASE		
lm	leptomeingeal disease	0	no
		1	yes
lmr	leptomeingeal disease at first recurrence	0	no
		1	yes
	TREATMENT		
sx	surgery	none	
		bx	biopsy
		str	subtotal resection
		gtr	gross total resection
		NA	not available
rt	radiotherapy	0	no
		1	yes
ct	chemotherapy	0	no
		1	yes
drt	details of radiotherapy	17 - 60	doses, Gy
		local	resection bed
		CSI	cranio-spinal irradiation
		SC	spinal cord
		SRSx	stereotactic radiosurgery
		NA	not available
		NaN	not a number i.e. no radiotherapy
dct	details of chemotherapy	ACNU	amino-chloroethyl nitrosourea
		ICE	ifosphamide, carboplatin, etoposide
		,	alternating
		VCiACy/E	vincristine, cisplatin, adriamycin, etoposide or cyclophosphamide
		VEICa	vincristine, etoposide, ifosfamide, carboplatin
		D	doxorubicin
		CV	cyclophosphamide, vincristine
		SJMB96	cisplatin, cyclophosphamide, vincristine + autologous bone marrow transplant
it	intrathecal chemotherapy	0	no
		1	yes (type unspecified)
		MTX	methotrexate
		LC	liposomal cytarabine
sx2	surgery at recurrence		
rt2	radiation at recurrence		
ct2	chemotherapy at recurrence		
	OUTCOME		
ttp	time to progression		months
p	progressed?	0	no
		1	yes
os	overall survival (time until last follow-up)		months

d	died?	0	no
	(at time of last follow-up)	1	yes
tpd	time from progression to death		months
	NOTES		
notes		PPx	prophylaxis
autopsy	autopsy performed	0	no
		1	yes
		NA	not available
			i.e. insufficient information
		NaN	not a number
			i.e. death not reported
	HISTOLOGY		
mib1	antigen KI-67/ MKI67	quantitative	1 to 90
		qualitative	"rare" to "extremely high"
del22q	vimentin (intermediate protein)	0	no
	mesenchymal/ immature cells	1	yes
		NA	not available
			i.e. insufficient information
		NaN	not a number
			i.e. not performed
			i.e. assumed not done if
			other stains were reported
ini1	integrase interactor 1	"	"
vim	vimentin	"	"
ema	epithelial membrane antigen	"	"
sma	smooth muscle actin	"	"
s100	100% soluble (in ammonium sulfate)	"	"
nfp	neurofilament protein	"	"
nse	neuron-specific enolase	"	"
cd56	neural cell adhesion molecule	"	"
nestin	nestin	"	"
olig2	oligodendrocyte transcription factor 2	"	"
gfap	glial fibrillary astrocytic protein	"	"
synaptophysin	major synaptic vesicle protein p38	"	"
chromograminA	parathyroid secretory protein 1	"	"
rcc	renal cell carcinoma antibody	"	"
cd57	B3GAT1	"	"
melanA	melanoma antigen	"	"
hmb45	human melanoma black 45	"	"
sox10	transcription factor SOX-10	"	"
EGFR	epidermal growth factor receptor	"	"
p63	transformation-related protein 63	"	"
brst2	GCDFP-15	"	"
mammaglobulin	mammaglobulin	"	"
cea	carcinoembryonic antigen	"	"
ck	cytokeratin [type not specified]	"	"
ck1	67 Da	"	"
ck2	65 Da	"	"
ck3	63 Da	"	"
ck4	59 Da	"	"
ck5	58 Da	"	"
ck6	56 Da	"	"

ck7	54 Da	" "
ck8	52 Da, CAM5.2	" "
ck9	64 Da	" "
ck10	56.5 Da	" "
ck11	56 Da	" "
ck12	55 Da	" "
ck13	51 Da	" "
ck14	50 Da	" "
ck15	50 Da	" "
ck16	48 Da	" "
ck17	46 Da	" "
ck18	45 Da	" "
ck19	40 Da	" "
ck20	46 Da	" "
ck26	type I inner root sheath-specific keratin	" "
desmin	desmin	" "
actin	actin	" "
sarcoActin	alpha-sarcometic actin	" "
cd31	PECAM-1	" "
myoglobin	myoglobin	" "
a1ACT	alpha1 anti-chymotrypsin	" "
a1AT	alpha1 anti-trypsin	" "
myogenin	Myf4	" "
cKit	CD117	" "
myosin	myosin	" "
glypican3	glypican-3	" "
MyoD1	Myogenic regulatory protein	" "
cd138	syndecan-1	" "
cd34	hematopoietic progenitor cell antigen CD34	" "
lca	leukocyte common antigen	" "
cd10	neprilysin	" "
cd99	single-chain type-1 glycoprotein	" "
cd3	CD3 T-cell co-receptor	" "
cd43	leukosialin	" "
cd20	B-lymphocyte antigen CD20	" "
vWF	von Willebrand Factor	" "
cd68	macrosialin	" "
cd1a	Leu6	" "
cd79a	MB-1 membrane glycoprotein	" "
plap	placental alkaline phosphatase	" "
hcg	human chorionic gonadotropin	" "
afp	alpha feto-protein	" "
inhibin	inhibin	" "
p53	cellular tumor antigen p53	" "
OCT3/4	octamer-binding transcription factor 3/4	" "
er	estrogen receptor	" "
pr	progesterone receptor	" "
mgmt	O-6 methylguanine DNA methyltransferase	" "
tab	transcription activator BRG1	" "

Table 1: Key to data

2 Data

```
dim(d1 <- data.table(read.csv("d1.csv", stringsAsFactors=FALSE)))

## [1]  50 108

d1 <- d1[, "ker" := 1 * (rowSums(.SD, na.rm=TRUE) > 0),
         .SDcols=grep("ck", colnames(d1))]
### uncomment below
### to remove cytokeratins from exploratory analysis
## ck1 <- grep("ck", colnames(d1))
## for (j in rev(ck1)) set(d1, i=NULL, j=j, value=NULL)
ihc1 <- names(d1)[29:ncol(d1)]
### missing data
sumNAOrNaN <- function (i) sum(!(is.na(i) | is.nan(i)))
s1 <- d1[, sapply(.SD, sumNAOrNaN),
   .SDcols=(length(d1) - length(ihc1)):length(d1)]
sum(s1 >= 3)

## [1] 50

sum(s1 >= 3) / length(ihc1) * 100

## [1] 61.7284

sum(s1 == 1)

## [1] 19

sum(s1 == 1) / length(ihc1) * 100

## [1] 23.45679

asFact <- function(x) factor(x,
                             levels=c(0, 1),
                             labels=c("neg", "pos"))
for (i in ihc1) {
  d1[, (i) := asFact(d1[[i]])]
}
### surgery as binary
d1 <- d1[!is.na(sx), sxb := !(sx=="none")]
d1 <- d1[!is.na(sx2), sxb2 := !(sx2=="none")]
d1 <- d1[!is.na(sx), gtr := sx=="gtr"]
d1 <- d1[!is.na(sx2), gtr2 := sx2=="gtr"]
d1 <- within(d1, {
  sex <- factor(sex, levels=c("m", "f"))
  csi <- as.logical(gregexpr("csi", drt, ignore.case=TRUE) > 1)
  ice <- grepl("ice", dct, ignore.case=TRUE)
  mib1n <- suppressWarnings(as.numeric(mib1))
  loc <- as.factor(loc)
  ## (super) short location
  ssloc <- sloc <- loc
  ## sx, rt and chemoTx
```

```
  src <- sxb * rt *ct
  sx <- factor(sx,
               levels=c("none", "bx", "str", "sx", "gtr"),
               ordered=TRUE)
})
### midline
d1 <- d1[!is.na(lat), midl := lat=="c"]
levels(d1$sloc) <- (c("4v", "4v", rep("lv", 4),
                      "4v", "pin", "pit", "lv", "pit", "cord",
                      "pit", "lv", "lv"))
levels(d1$ssloc) <- (c("bs", "bs", rep("lv", 4),
                      "bs", "lv", "bs", "lv", "bs", "cord",
                       "bs", "lv", "lv"))
length(table(d1$loc))

## [1] 15

length(table(d1$sloc))

## [1] 5

length(table(d1$ssloc))

## [1] 3

asFact <- function(x) factor(x,
                             levels=c(0, 1),
                             labels=c("no", "yes"))
for (i in c("fert", "preg",
            "csf", "lm", "lmr",
            "rt", "ct",
            "rt2", "ct2")) {
  d1[, (i) := asFact(d1[[i]])]
}
d1 <- d1[!is.na(it), itl := !(it==0)]
```

3 Useful functions

```
suppressPackageStartupMessages(library("survMisc"))
xtable.table <- survMisc:::xtable.table
xtable.survfit <- survMisc:::xtable.survfit
xtable <- xtable::xtable
### generalized correlation function
assoc <- function(x, y) {
  stopifnot(length(x)==length(y))
  df1 <- data.frame(x, y)
  c1 <- sapply(df1, class)
  if (any(c1=="logical")) {
    df1[which(c1=="logical")] <- as.factor(df1[[which(c1=="logical")]])
  }
```

```
  c1 <- sapply(df1, class)
  if (all(sapply(c1, function(i) "numeric" %in% i))) s1 <- "n"
  if (all(sapply(c1, function(i) "factor" %in% i))) {
    s1 <- "f"
  } else {
    s1 <- "nf"
  }
  if ("character" %in% c1) s1 <- "c"
  res1 <- switch(s1,
                 n=cont2(x=x, y=y),
                 f=fact2(x=x, y=y),
                 nf=contFact(x=x, y=y),
                 c=c("char", NaN, NaN, NaN)
                 )
  return(res1)
}
cont2 <- function(x, y, sigD=2){
  res1 <- c("Pearsons r",
            signif(cor(x, y), sigD),
            "t-test",
            signif(t.test(x, y)$p.value), sigD)
  return(res1)
}
fact2 <- function(x, y, sigD=2) {
  t1 <- table(x, y)
  n <- sum(t1)
  sr <- rowSums(t1)
  sc <- colSums(t1)
  E <- outer(sr, sc, "*") / sum(t1)
  stat1 <- sum((abs(t1 - E))^2 / E)
  df1 <- (nrow(t1) - 1L) * (ncol(t1) - 1L)
  cr1 <- signif(
    sqrt(stat1 /
        (length(complete.cases(x, y) *
                (min(nlevels(x),
                     nlevels(y)) - 1)))), sigD)
  c1 <- signif(stats::pchisq(stat1, df1, lower.tail=FALSE), sigD)
  res1 <- c("Cramers V",
            cr1,
            "Chi-squared",
            c1)
  return(res1)
}
### function imported below
suppressPackageStartupMessages(library("heplots"))
contFact <- function(x, y, sigD=2) {
  df1 <- data.frame(x, y)
  s1 <- sapply(df1, is.numeric)
  df1 <- df1[c(which(s1), which(!s1))]
  a1 <- tryCatch(aov(df1[[1]] ~ df1[[2]]),
                 error=function(e) e)
```

```
  res1 <- tryCatch(c("Pearsons r",
                     signif(sqrt(heplots::etasq(a1)["df1", ]), sigD),
                     "F test",
                     signif(unlist(summary(a1))[c("Pr(>F)1")], sigD)),
                   error=function(e)
                   c("Pearsons r", NaN, "F test", NaN))
  return(res1)
}
```

4 Missing data

```
m1 <- apply(is.na(d1), 2, sum)
round(sum(m1 > nrow(d1) / 2) / length(m1) * 100, 1)
```

[1] 64.5

```
m1 <- sort(round(m1 / nrow(d1) * 100, 1))
m1 <- cbind(names(m1), m1)
m1 <- cbind(m1[1:(nrow(m1) / 2), ],
            m1[(nrow(m1) / 2 + 1):nrow(m1), ])
colnames(m1) <- rep(c("variable", "% missing"), times=2)
options("xtable.include.rownames"=FALSE)
print(xtable(m1,
             caption="Missing values (percentages)",
             align=c("c", rep(c("r", "l"), times=2))),
      tabular.environment="longtable",
      floating=FALSE)
```

variable	% missing	variable	% missing
first	0	ck5	80
yr	0	ck6	80
age	0	ck19	80
sex	0	hmb45	82
fert	0	ck10	82
loc	0	ck14	82
ker	0	ck15	82
ssloc	0	ck16	82
sloc	0	lca	84
ice	0	actin	86
lat	4	cd31	86
midl	4	cd99	86
os	8	nse	88
d	8	melanA	90
lm	10	p53	90
csf	14	cd56	92
sx	14	ck17	92
sxb	14	ck18	92
gtr	14	myoglobin	92

rt	16	plap	92
ct	16	hcg	92
src	16	afp	92
it	18	ck20	94
sx2	18	a1ACT	94
sxb2	18	cKit	94
gtr2	18	cd138	94
itl	18	vWF	94
autopsy	20	er	94
mib1	26	pr	94
p	30	nestin	96
ema	30	olig2	96
drt	32	p63	96
dct	32	brst2	96
csi	32	mammaglobulin	96
ttp	34	ck12	96
vim	34	ck13	96
lmr	36	sarcoActin	96
sma	36	myogenin	96
gfap	40	MyoD1	96
tpd	46	cd10	96
s100	46	mgmt	96
rt2	48	rcc	98
ct2	48	cd57	98
ini1	52	sox10	98
synaptophysin	56	EGFR	98
ck	56	cea	98
preg	60	ck11	98
notes	60	ck26	98
desmin	60	a1AT	98
ck8	68	myosin	98
del22q	70	glypican3	98
nfp	70	cd3	98
ck7	76	cd43	98
cd34	78	cd20	98
mib1n	78	cd68	98
chromograminA	80	cd1a	98
ck1	80	cd79a	98
ck2	80	inhibin	98
ck3	80	OCT3.4	98
ck4	80	tab	98

Table 2: Missing values (percentages)

5 Descriptives

```
options("xtable.include.rownames"=TRUE)
m1 <- matrix(
  prettyNum(signif(contr.poly(5), 2)),
  nrow=5,
```

```
  dimnames=list(
    levels(d1$sx),
    paste0("x^", seq(1:4))))
xtable(t(m1),
       caption="Sx as ordinal; polynomial contrasts")
```

	none	bx	str	sx	gtr
x^1	-0.63	-0.32	0	0.32	0.63
x^2	0.53	-0.27	-0.53	-0.27	0.53
x^3	-0.32	0.63	-4.1e-16	-0.63	0.32
x^4	0.12	-0.48	0.72	-0.48	0.12

Table 3: Sx as ordinal; polynomial contrasts

```
xtable(d1[, table("location"=loc, "location (5 groups)"=sloc)])
```

	location (5 groups)	.	.	.	.
location	4v	lv	pin	pit	cord
cb	3	0	0	0	0
cpa	3	0	0	0	0
f	0	6	0	0	0
fp	0	2	0	0	0
o	0	2	0	0	0
pa	0	5	0	0	0
pf	1	0	0	0	0
pi	0	0	6	0	0
pit	0	0	0	1	0
pt	0	1	0	0	0
s	0	0	0	6	0
sc	0	0	0	0	6
ss	0	0	0	4	0
t	0	3	0	0	0
th	0	1	0	0	0

Table 4: location × location (5 groups)
chi-sq=4.7e-18

```
xtable(d1[, table("location"=loc, "location (3 groups)"=ssloc)])
```

	location (3 groups)	.	.
location	bs	lv	cord
cb	3	0	0
cpa	3	0	0
f	0	6	0
fp	0	2	0
o	0	2	0
pa	0	5	0
pf	1	0	0
pi	0	6	0
pit	1	0	0
pt	0	1	0
s	6	0	0
sc	0	0	6
ss	4	0	0
t	0	3	0
th	0	1	0

Table 5: location × location (3 groups)
chi-sq=5.1e-10

```
options("xtable.include.rownames"=FALSE)
xtable(t(as.matrix(summary(d1$age))),
       caption="Age; Qu. = quantile")
```

Min.	1st Qu.	Median	Mean	3rd Qu.	Max.
18.00	23.00	31.50	33.68	42.00	65.00

Table 6: Age; Qu. = quantile

```
options("xtable.include.rownames"=TRUE)
xtable(table(d1$sex))
```

	m	f
.	23	27

Table 7:
chi-sq=0.57

```
xtable(table(d1$preg),
       caption="Pregnant?")
```

	no	yes
.	17	3

Table 8: Pregnant?

```
options("xtable.include.rownames"=TRUE)
s1 <- seq(from=1, to=length(ihc1), by=10)
for (i in 1:(length(s1) - 1)) {
  print(
    xtable(
      d1[, sapply(.SD, table),
         .SDcols=ihc1[s1[i]:(s1[i+1] - 1)]]))
}
```

	del22q	ini1	vim	ema	sma	s100	nfp	nse	cd56	nestin
neg	2	23	0	6	14	14	5	4	3	0
pos	13	1	33	29	18	13	10	2	1	2

	olig2	gfap	synaptophysin	chromograminA	rcc	cd57	melanA	hmb45	sox10	EGFR
neg	1	18	16	9	1	1	4	9	1	1
pos	1	12	6	1	0	0	1	0	0	0

	p63	brst2	mammaglobulin	cea	ck	ck1	ck2	ck3	ck4	ck5
neg	2	2	2	1	10	5	5	5	5	5
pos	0	0	0	0	12	5	5	5	5	5

	ck6	ck7	ck8	ck9	ck10	ck11	ck12	ck13	ck14	ck15
neg	5	7	4	0	5	1	1	1	5	5
pos	5	5	12	0	4	0	1	1	4	4

	ck16	ck17	ck18	ck19	ck20	ck26	desmin	actin	sarcoActin	cd31
neg	5	2	2	5	3	1	19	3	2	7
pos	4	2	2	5	0	0	1	4	0	0

	myoglobin	a1ACT	a1AT	myogenin	cKit	myosin	glypican3	MyoD1	cd138	cd34
neg	4	1	0	2	3	1	0	2	3	8
pos	0	2	1	0	0	0	1	0	0	3

	lca	cd10	cd99	cd3	cd43	cd20	vWF	cd68	cd1a	cd79a
neg	8	2	5	1	1	1	3	0	1	1
pos	0	0	2	0	0	0	0	1	0	0

	plap	hcg	afp	inhibin	p53	OCT3.4	er	pr	mgmt	tab
neg	4	4	4	1	1	1	3	3	0	1
pos	0	0	0	0	4	0	0	0	2	0

```
d1[, sum(!is.na(mib1) & !mib1=="NaN")]
```

[1] 25

```
xtable(d1[, table(mib1n)])
```

	1	5.8	8.4	13.5	30	40	45	55	80	90
.	1	1	1	1	2	1	1	1	1	1

Table 9: mib1n
chi-sq=1

```
plot(d1[, sort(mib1n) / 100],
     type="b", ylim=0:1,
     main="MIB-1 close to exponential with unit slope")
abline(0, 1 /(10+1), col=adjustcolor(1, 0.5))
r1 <- d1[!is.na(mib1n),
         fitdistr(mib1n / 100, "exponential")]$estimate

## Error in eval(expr, envir, enclos):  could not find function "fitdistr"

qqplot(qexp(ppoints(11)),
       d1[, sort(mib1n)],
       main=expression("Quantile-Quantile plot: Exponential vs. MIB-1"),
       xlab="Exponential",
       ylab="MIB-1")
qqline(d1[, sort(mib1n)],
       distribution = qexp,
       probs=c(0.1, 0.8),
       col=2)
plot(d1[, sort(log(mib1n))],
     type="b",
     main="ln (MIB-1) is approximately linear",
     ylab="ln (MIB-1)")
abline(0, 0.5, col="red")
xtable(d1[, table(lm, useNA="ifany")])
```

	no	yes	NA
.	35	10	5

Table 10: lm
chi-sq=1.9e-07

```
xtable(d1[, table(lmr, useNA="ifany")])
```

	no	yes	NA
.	20	12	18

Table 11: lmr
chi-sq=0.35

```
xtable(d1[, table(csf, useNA="ifany")])
```

	no	yes	NA
.	3	40	7

Table 12: csf
chi-sq=1.8e-11

```
xtable(d1[, table(sloc, useNA="ifany")])
```

	4v	lv	pin	pit	cord
.	7	20	6	11	6

Table 13: sloc
chi-sq=0.0067

```
xtable(d1[, table(ssloc, useNA="ifany")])
```

	bs	lv	cord
.	18	26	6

Table 14: ssloc
chi-sq=0.0023

```
xtable(d1[, table(lat, useNA="ifany")])
```

	c	l	r	NA
.	24	11	13	2

Table 15: lat
chi-sq=0.00021

```
xtable(d1[, table(midl, useNA="ifany")])
```

	FALSE	TRUE	NA
.	24	24	2

Table 16: midl
chi-sq=6.3e-05

```
xtable(d1[, table(autopsy)])
```

	0	1
.	38	2

Table 17: autopsy
chi-sq=1.3e-08

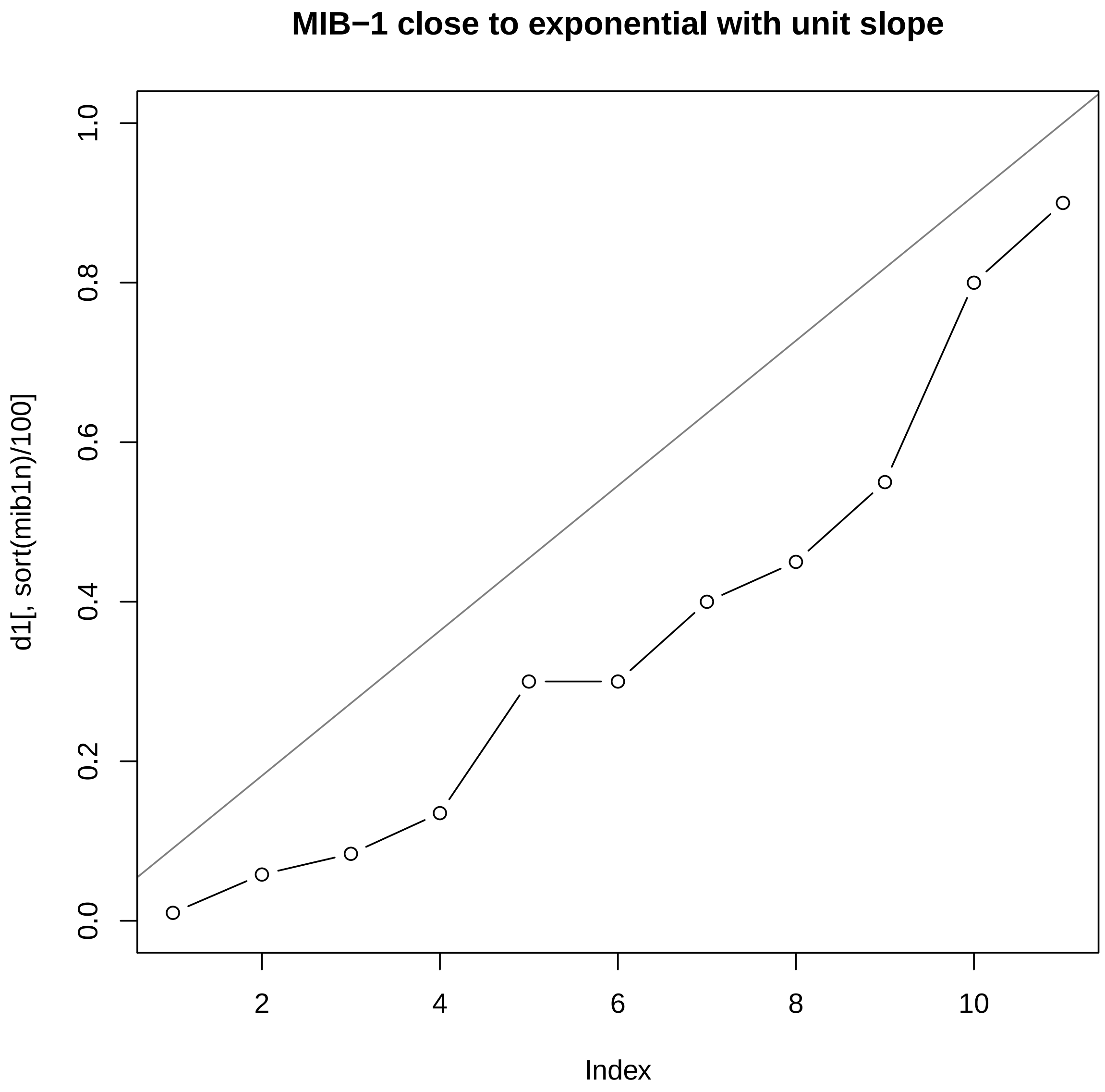
MIB−1 close to exponential with unit slope
d1[, sort(mib1n)/100]
0.0
0.2
0.4
0.6
0.8
1.0
2
4
6
8
10
Index

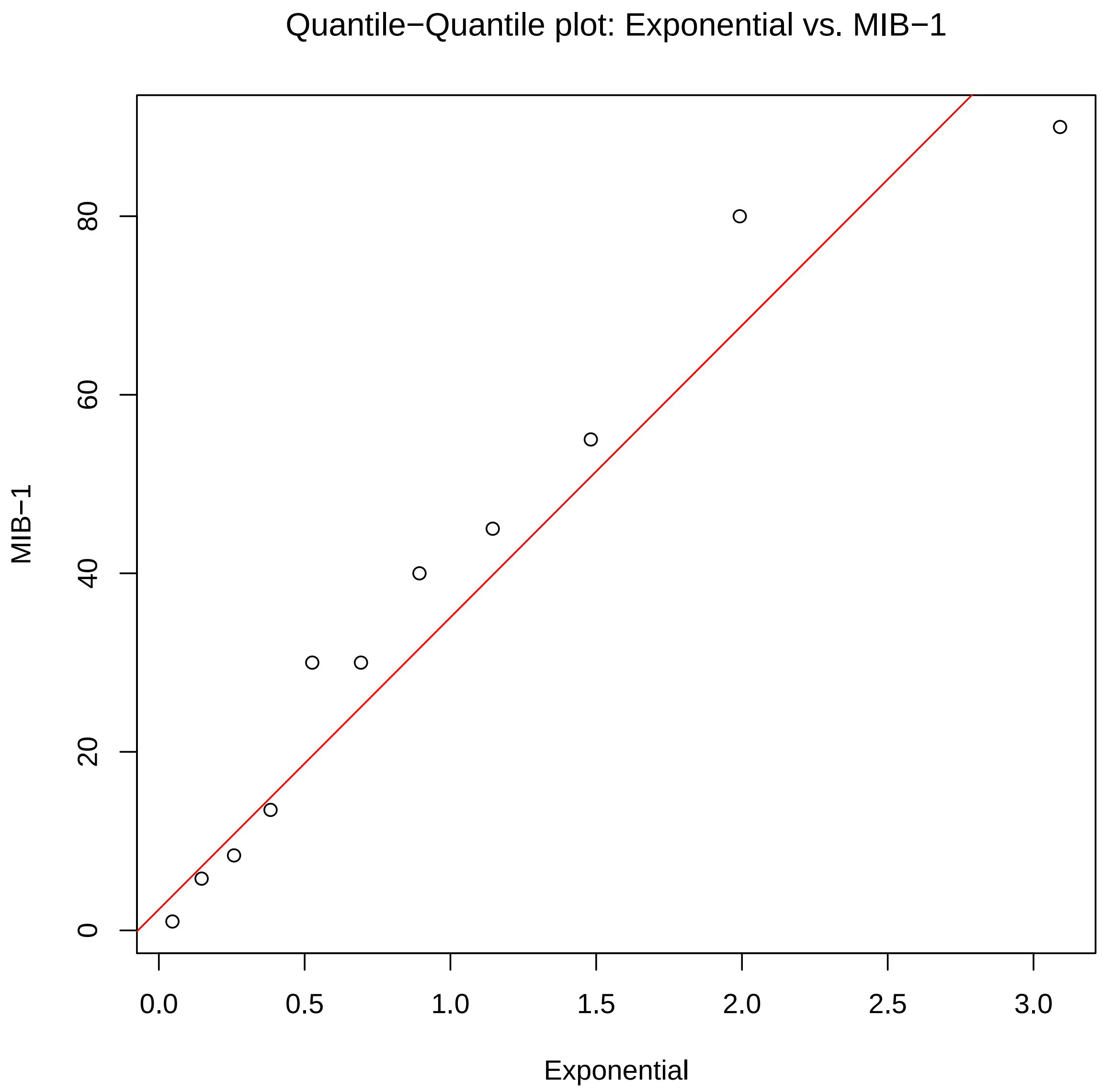
Quantile−Quantile plot: Exponential vs. MIB−1
MIB−1
0
20
40
60
80
0.0
0.5
1.0
1.5
2.0
2.5
3.0
Exponential

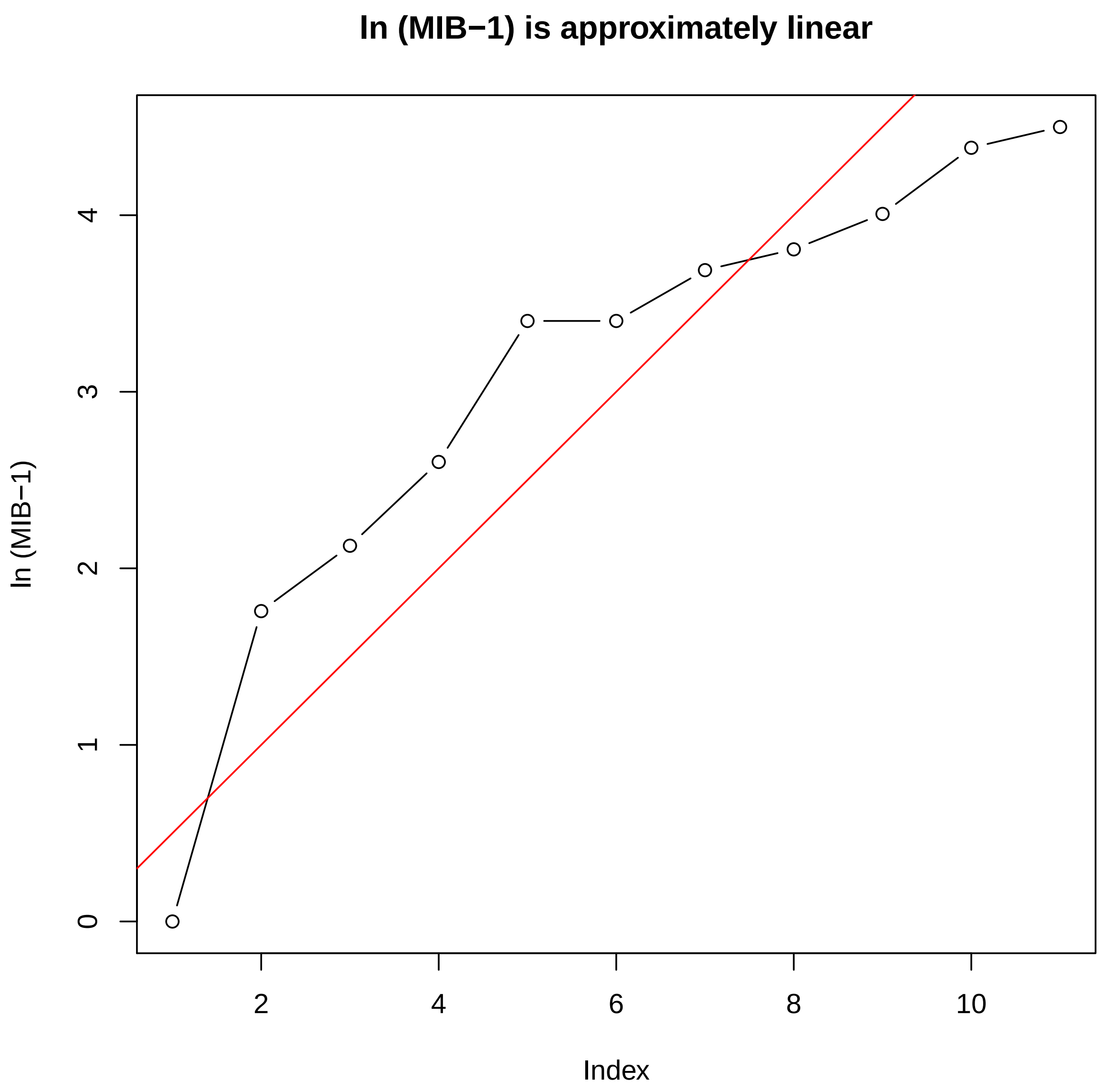

6 Outcomes

```
options("xtable.include.rownames"=FALSE)
suppressPackageStartupMessages(library("survival"))
xtable(survfit(Surv(ttp, as.numeric(p)) ~ 1, data=d1),
       caption="Time to progression")
```

records	n.max	n.start	events	median	0.95LCL	0.95UCL
33	33	33	29	5	3	18

Table 18: Time to progression

```
xtable(survfit(Surv(os, as.numeric(d)) ~ 1, data=d1),
       caption="Overall survival")
```

records	n.max	n.start	events	median	0.95LCL	0.95UCL
46	46	46	30	23	14	56

Table 19: Overall survival

```
xtable(survfit(Surv(tpd, as.numeric(d)) ~ 1, data=d1),
       caption="Time from progression to death")
```

records	n.max	n.start	events	median	0.95LCL	0.95UCL
27	27	27	22	8	5	28

Table 20: Time from progression to death

```
options("width"=100)
summary(survfit(Surv(ttp, as.numeric(p)) ~ 1, data=d1),
        times=c(1, 6, 12, 24))

## Call: survfit(formula = Surv(ttp, as.numeric(p)) ~ 1, data = d1)
##
## 17 observations deleted due to missingness
##  time n.risk n.event survival std.err lower 95% CI upper 95% CI
##     1     32       5    0.848  0.0624        0.735        0.980
##     6     16      13    0.455  0.0867        0.313        0.661
##    12     13       4    0.333  0.0821        0.206        0.540
##    24      7       4    0.202  0.0715        0.101        0.404

summary(survfit(Surv(os, as.numeric(d)) ~ 1, data=d1),
        times=c(1, 6, 12, 24))

## Call: survfit(formula = Surv(os, as.numeric(d)) ~ 1, data = d1)
##
## 4 observations deleted due to missingness
##  time n.risk n.event survival std.err lower 95% CI upper 95% CI
##     1     46       0    1.000  0.0000        1.000        1.000
##     6     40      11    0.756  0.0641        0.640        0.892
##    12     27       5    0.643  0.0716        0.517        0.800
##    24     16       9    0.391  0.0794        0.262        0.582

summary(survfit(Surv(tpd, as.numeric(d)) ~ 1, data=d1),
        times=c(1, 6, 12, 24))

## Call: survfit(formula = Surv(tpd, as.numeric(d)) ~ 1, data = d1)
##
## 23 observations deleted due to missingness
##  time n.risk n.event survival std.err lower 95% CI upper 95% CI
```

```
##      1      24         5     0.809  0.0769          0.671          0.975
##      6      16         8     0.501  0.0981          0.341          0.735
##     12      10         2     0.420  0.0975          0.267          0.662
##     24       6         3     0.272  0.0939          0.139          0.535

options("width"=80)
d1[d==0 & os > 36, first]

## [1] "Makuria"   "Takahashi" "Dardis"
```

7 Treatment

```
## names(d1)
## names(d1)[c(12:20, 110:114, 117:119)]
s1 <- c(12:20, 110:114, 117:119)
for (i in s1) {
  print(names(d1)[i])
  print(
    xtable(
      d1[, as.table(sort(table(.SD), decreasing=TRUE)), .SDcols=i]))}
```

[1] "sx"

str	gtr	sx	bx	none
21	10	8	3	1

Table 21: .SD
chi-sq=9.8e-06

[1] "rt"

yes	no
33	9

Table 22: .SD
chi-sq=0.00021

[1] "ct"

no	yes
25	17

Table 23: .SD
chi-sq=0.22

[1] "it"

0	LC x6	MTX x1	MTX x3
38	1	1	1

Table 24: .SD
chi-sq=1.4e-21

[1] "drt"

54 local	60 local	17 SRSx	50 local	cranial	CSI	30 local, 30 CSI	50 local, 30 CSI	53 local, 35 CSI	54 local +
4	4	2	2	2	2	1	1	1	1

Table 25: .SD
chi-sq=0.82

[1] "dct"

ICE x6	5 agent	ACNU	D, ICE x3, CV x5	ICE	ICE x2	ICE x8	NAN	SJMB96 x4	VCiACy/E, VEICa
2	1	1	1	1	1	1	1	1	1

Table 26: .SD
chi-sq=1

[1] "sx2"

none	gtr	str	sx
14	5	4	3

Table 27: .SD
chi-sq=0.0079

[1] "rt2"

no	yes
13	13

Table 28: .SD
chi-sq=1

[1] "ct2"

no	yes
15	11

Table 29: .SD
chi-sq=0.43

[1] "sxb"

TRUE	FALSE
42	1

Table 30: .SD
chi-sq=4e-10

[1] "sxb2"

TRUE	FALSE
27	14

Table 31: .SD
chi-sq=0.042

[1] "gtr"

FALSE	TRUE
33	10

Table 32: .SD
chi-sq=0.00045

[1] "gtr2"

FALSE	TRUE
36	5

Table 33: .SD
chi-sq=1.3e-06

[1] "src"

0	1
27	15

Table 34: .SD
chi-sq=0.064

[1] "mib1n"

30	1	5.8	8.4	13.5	40	45	55	80	90
2	1	1	1	1	1	1	1	1	1

Table 35: .SD
chi-sq=1

[1] "ice"

FALSE	TRUE
44	6

Table 36: .SD
chi-sq=7.7e-08

[1] "csi"

FALSE	TRUE
29	5

Table 37: .SD
chi-sq=3.9e-05

```
options("xtable.include.rownames"=TRUE)
xtable(d1[, table("surgery"=sx, "short location"=sloc)])
```

	short location	.	.	.	.
surgery	4v	lv	pin	pit	cord
none	1	0	0	0	0
bx	0	2	0	0	1
str	2	5	4	7	3
sx	2	4	0	2	0
gtr	1	7	1	1	0

Table 38: surgery × short location
chi-sq=0.2

8 Exploratory analysis; binary associations

```
c1 <- combn(as.data.frame(d1), 2, simplify=FALSE)
names(c1) <- lapply(c1, function(x) paste0(names(x)))
suppressWarnings(
  a1 <- sapply(c1, function(x) assoc(x=x[, 1],
                                      y=x[, 2])))
a1 <- as.data.frame(t(a1))
## sort by significance
a1 <- a1[order(a1$Pr), ]
with(options(width=100),
     head(a1, 250))

##                                            V1   V2          V3 Pr(>F)1
## c("dct", "src")                    Pearsons r    1      F test       0
## c("lat", "loc")                     Cramers V  1.1 Chi-squared 0.00014
## c("src", "ice")                    Pearsons r 0.55      F test 0.00017
## c("it", "ice")                      Cramers V 0.61 Chi-squared 0.00029
## c("loc", "midl")                    Cramers V 0.89 Chi-squared 0.00032
```

```
## c("sx2", "p")                          Pearsons r 0.72      F test 0.00035
## c("ct", "d")                           Pearsons r 0.54      F test 0.00036
## c("p", "csi")                          Pearsons r 0.63      F test 0.00036
## c("p", "src")                          Pearsons r 0.57      F test 0.00037
## c("ssloc", "midl")                      Cramers V 0.55 Chi-squared 0.00047
## c("p", "d")                            Pearsons r 0.56      F test 0.00064
## c("age", "del22q")                     Pearsons r 0.77      F test 0.00087
## c("csf", "gtr")                         Cramers V 0.47 Chi-squared   0.001
## c("ttp", "os")                         Pearsons r 0.56      F test  0.0011
## c("drt", "src")                        Pearsons r 0.89      F test  0.0012
## c("ct", "ice")                          Cramers V 0.45 Chi-squared  0.0013
## c("ck1", "ck2")                         Cramers V 0.45 Chi-squared  0.0016
## c("ck1", "ck3")                         Cramers V 0.45 Chi-squared  0.0016
## c("ck1", "ck4")                         Cramers V 0.45 Chi-squared  0.0016
## c("ck1", "ck5")                         Cramers V 0.45 Chi-squared  0.0016
## c("ck1", "ck6")                         Cramers V 0.45 Chi-squared  0.0016
## c("ck1", "ck7")                         Cramers V 0.45 Chi-squared  0.0016
## c("ck2", "ck3")                         Cramers V 0.45 Chi-squared  0.0016
## c("ck2", "ck4")                         Cramers V 0.45 Chi-squared  0.0016
## c("ck2", "ck5")                         Cramers V 0.45 Chi-squared  0.0016
## c("ck2", "ck6")                         Cramers V 0.45 Chi-squared  0.0016
## c("ck2", "ck7")                         Cramers V 0.45 Chi-squared  0.0016
## c("ck3", "ck4")                         Cramers V 0.45 Chi-squared  0.0016
## c("ck3", "ck5")                         Cramers V 0.45 Chi-squared  0.0016
## c("ck3", "ck6")                         Cramers V 0.45 Chi-squared  0.0016
## c("ck3", "ck7")                         Cramers V 0.45 Chi-squared  0.0016
## c("ck4", "ck5")                         Cramers V 0.45 Chi-squared  0.0016
## c("ck4", "ck6")                         Cramers V 0.45 Chi-squared  0.0016
## c("ck4", "ck7")                         Cramers V 0.45 Chi-squared  0.0016
## c("ck5", "ck6")                         Cramers V 0.45 Chi-squared  0.0016
## c("ck5", "ck7")                         Cramers V 0.45 Chi-squared  0.0016
## c("ck6", "ck7")                         Cramers V 0.45 Chi-squared  0.0016
## c("ct", "p")                           Pearsons r  0.5      F test  0.0024
## c("p", "desmin")                       Pearsons r 0.68      F test  0.0024
## c("ck1", "ck10")                        Cramers V 0.42 Chi-squared  0.0027
## c("ck1", "ck14")                        Cramers V 0.42 Chi-squared  0.0027
## c("ck1", "ck15")                        Cramers V 0.42 Chi-squared  0.0027
## c("ck1", "ck16")                        Cramers V 0.42 Chi-squared  0.0027
## c("ck1", "ck19")                        Cramers V 0.42 Chi-squared  0.0027
## c("ck2", "ck10")                        Cramers V 0.42 Chi-squared  0.0027
## c("ck2", "ck14")                        Cramers V 0.42 Chi-squared  0.0027
## c("ck2", "ck15")                        Cramers V 0.42 Chi-squared  0.0027
## c("ck2", "ck16")                        Cramers V 0.42 Chi-squared  0.0027
## c("ck2", "ck19")                        Cramers V 0.42 Chi-squared  0.0027
## c("ck3", "ck10")                        Cramers V 0.42 Chi-squared  0.0027
## c("ck3", "ck14")                        Cramers V 0.42 Chi-squared  0.0027
## c("ck3", "ck15")                        Cramers V 0.42 Chi-squared  0.0027
## c("ck3", "ck16")                        Cramers V 0.42 Chi-squared  0.0027
## c("ck3", "ck19")                        Cramers V 0.42 Chi-squared  0.0027
## c("ck4", "ck10")                        Cramers V 0.42 Chi-squared  0.0027
## c("ck4", "ck14")                        Cramers V 0.42 Chi-squared  0.0027
```

```
## c("ck4", "ck15")                        Cramers V 0.42 Chi-squared  0.0027
## c("ck4", "ck16")                        Cramers V 0.42 Chi-squared  0.0027
## c("ck4", "ck19")                        Cramers V 0.42 Chi-squared  0.0027
## c("ck5", "ck10")                        Cramers V 0.42 Chi-squared  0.0027
## c("ck5", "ck14")                        Cramers V 0.42 Chi-squared  0.0027
## c("ck5", "ck15")                        Cramers V 0.42 Chi-squared  0.0027
## c("ck5", "ck16")                        Cramers V 0.42 Chi-squared  0.0027
## c("ck5", "ck19")                        Cramers V 0.42 Chi-squared  0.0027
## c("ck6", "ck10")                        Cramers V 0.42 Chi-squared  0.0027
## c("ck6", "ck14")                        Cramers V 0.42 Chi-squared  0.0027
## c("ck6", "ck15")                        Cramers V 0.42 Chi-squared  0.0027
## c("ck6", "ck16")                        Cramers V 0.42 Chi-squared  0.0027
## c("ck6", "ck19")                        Cramers V 0.42 Chi-squared  0.0027
## c("ck7", "ck10")                        Cramers V 0.42 Chi-squared  0.0027
## c("ck7", "ck14")                        Cramers V 0.42 Chi-squared  0.0027
## c("ck7", "ck15")                        Cramers V 0.42 Chi-squared  0.0027
## c("ck7", "ck16")                        Cramers V 0.42 Chi-squared  0.0027
## c("ck10", "ck14")                       Cramers V 0.42 Chi-squared  0.0027
## c("ck10", "ck15")                       Cramers V 0.42 Chi-squared  0.0027
## c("ck10", "ck16")                       Cramers V 0.42 Chi-squared  0.0027
## c("ck10", "ck19")                       Cramers V 0.42 Chi-squared  0.0027
## c("ck14", "ck15")                       Cramers V 0.42 Chi-squared  0.0027
## c("ck14", "ck16")                       Cramers V 0.42 Chi-squared  0.0027
## c("ck14", "ck19")                       Cramers V 0.42 Chi-squared  0.0027
## c("ck15", "ck16")                       Cramers V 0.42 Chi-squared  0.0027
## c("ck15", "ck19")                       Cramers V 0.42 Chi-squared  0.0027
## c("ck16", "ck19")                       Cramers V 0.42 Chi-squared  0.0027
## c("yr", "sx")                          Pearsons r 0.58      F test  0.0029
## c("sx2", "d")                          Pearsons r 0.61      F test  0.0032
## c("sex", "ct2")                         Cramers V 0.42 Chi-squared  0.0033
## c("lat", "ssloc")                       Cramers V 0.56 Chi-squared  0.0034
## c("src", "sloc")                       Pearsons r 0.58      F test  0.0034
## c("preg", "itl")                        Cramers V 0.41 Chi-squared  0.0036
## c("os", "notes")                       Pearsons r 0.99      F test  0.0044
## c("age", "ssloc")                      Pearsons r 0.45      F test  0.0045
## c("csf", "gtr2")                        Cramers V  0.4 Chi-squared   0.005
## c("fert", "ct2")                        Cramers V 0.39 Chi-squared  0.0055
## c("lm", "gfap")                         Cramers V 0.39 Chi-squared  0.0057
## c("ttp", "p53")                        Pearsons r 0.97      F test  0.0061
## c("age", "sloc")                       Pearsons r 0.52      F test  0.0062
## c("d", "ice")                          Pearsons r 0.39      F test  0.0066
## c("yr", "notes")                       Pearsons r 0.98      F test  0.0071
## c("ct", "sloc")                         Cramers V 0.53 Chi-squared  0.0079
## c("loc", "src")                        Pearsons r 0.78      F test  0.0081
## c("sma", "synaptophysin")               Cramers V 0.37 Chi-squared  0.0081
## c("ini1", "ema")                        Cramers V 0.37 Chi-squared  0.0083
## c("csf", "d")                          Pearsons r 0.41      F test  0.0085
## c("ck7", "ck19")                        Cramers V 0.37 Chi-squared  0.0098
## c("ttp", "d")                          Pearsons r 0.46      F test  0.0099
## c("yr", "lat")                         Pearsons r 0.43      F test   0.011
## c("rt", "src")                         Pearsons r 0.39      F test   0.011
```

```
## c("sx2", "src")                      Pearsons r 0.55      F test   0.011
## c("yr", "sex")                       Pearsons r 0.35      F test   0.013
## c("csf", "ttp")                      Pearsons r 0.45      F test   0.013
## c("sx2", "autopsy")                  Pearsons r 0.58      F test   0.013
## c("src", "csi")                      Pearsons r 0.42      F test   0.013
## c("sx", "gtr2")                       Cramers V  0.5 Chi-squared   0.014
## c("ct", "nse")                        Cramers V 0.35 Chi-squared   0.014
## c("ck", "ck8")                        Cramers V 0.35 Chi-squared   0.014
## c("it", "p")                         Pearsons r 0.48      F test   0.015
## c("synaptophysin", "ice")             Cramers V 0.34 Chi-squared   0.015
## c("ssloc", "csi")                     Cramers V 0.41 Chi-squared   0.015
## c("s100", "gfap")                     Cramers V 0.34 Chi-squared   0.017
## c("src", "itl")                      Pearsons r 0.37      F test   0.017
## c("yr", "rt")                        Pearsons r 0.36      F test   0.018
## c("ema", "sxb2")                      Cramers V 0.33 Chi-squared   0.019
## c("sex", "midl")                      Cramers V 0.33 Chi-squared    0.02
## c("rt", "ema")                        Cramers V 0.33 Chi-squared   0.021
## c("lat", "sx")                        Cramers V  0.6 Chi-squared   0.022
## c("yr", "midl")                      Pearsons r 0.33      F test   0.024
## c("dct", "d")                        Pearsons r 0.74      F test   0.024
## c("sex", "nfp")                       Cramers V 0.32 Chi-squared   0.025
## c("lmr", "cd99")                      Cramers V 0.32 Chi-squared   0.025
## c("ct", "p53")                        Cramers V 0.32 Chi-squared   0.025
## c("nfp", "chromograminA")             Cramers V 0.32 Chi-squared   0.025
## c("ck", "ck19")                       Cramers V 0.32 Chi-squared   0.025
## c("ct", "rt2")                        Cramers V 0.31 Chi-squared   0.027
## c("tpd", "ema")                      Pearsons r 0.47      F test   0.027
## c("ct2", "ck1")                       Cramers V 0.31 Chi-squared   0.028
## c("ct2", "ck2")                       Cramers V 0.31 Chi-squared   0.028
## c("ct2", "ck3")                       Cramers V 0.31 Chi-squared   0.028
## c("ct2", "ck4")                       Cramers V 0.31 Chi-squared   0.028
## c("ct2", "ck5")                       Cramers V 0.31 Chi-squared   0.028
## c("ct2", "ck6")                       Cramers V 0.31 Chi-squared   0.028
## c("ct2", "ck19")                      Cramers V 0.31 Chi-squared   0.028
## c("yr", "rt2")                       Pearsons r 0.43      F test   0.029
## c("sex", "p")                        Pearsons r 0.37      F test   0.029
## c("csf", "sx")                        Cramers V 0.47 Chi-squared   0.029
## c("ct", "itl")                        Cramers V  0.3 Chi-squared   0.033
## c("yr", "chromograminA")             Pearsons r 0.67      F test   0.035
## c("age", "loc")                      Pearsons r 0.68      F test   0.035
## c("drt", "csi")                       Cramers V 0.71 Chi-squared   0.035
## c("rt2", "nfp")                       Cramers V  0.3 Chi-squared   0.035
## c("lat", "gtr")                       Cramers V 0.36 Chi-squared   0.037
## c("sx", "sx2")                        Cramers V 0.66 Chi-squared   0.037
## c("ct", "csi")                        Cramers V 0.29 Chi-squared   0.037
## c("loc", "ct")                        Cramers V  0.7 Chi-squared   0.038
## c("ck1", "ck8")                       Cramers V 0.29 Chi-squared   0.038
## c("ck1", "ker")                       Cramers V 0.29 Chi-squared   0.038
## c("ck2", "ck8")                       Cramers V 0.29 Chi-squared   0.038
## c("ck2", "ker")                       Cramers V 0.29 Chi-squared   0.038
## c("ck3", "ck8")                       Cramers V 0.29 Chi-squared   0.038
```

```
## c("ck3", "ker")                          Cramers V 0.29 Chi-squared   0.038
## c("ck4", "ck8")                          Cramers V 0.29 Chi-squared   0.038
## c("ck4", "ker")                          Cramers V 0.29 Chi-squared   0.038
## c("ck5", "ck8")                          Cramers V 0.29 Chi-squared   0.038
## c("ck5", "ker")                          Cramers V 0.29 Chi-squared   0.038
## c("ck6", "ck8")                          Cramers V 0.29 Chi-squared   0.038
## c("ck6", "ker")                          Cramers V 0.29 Chi-squared   0.038
## c("ck8", "ck19")                         Cramers V 0.29 Chi-squared   0.038
## c("ck19", "ker")                         Cramers V 0.29 Chi-squared   0.038
## c("lmr", "autopsy")                     Pearsons r  0.4      F test   0.039
## c("lmr", "ssloc")                        Cramers V 0.36 Chi-squared   0.039
## c("yr", "del22q")                       Pearsons r 0.53      F test    0.04
## c("csf", "sx2")                          Cramers V 0.41 Chi-squared    0.04
## c("p", "mib1")                          Pearsons r  0.9      F test   0.042
## c("yr", "src")                          Pearsons r 0.31      F test   0.043
## c("fert", "midl")                        Cramers V 0.29 Chi-squared   0.043
## c("rt", "ct")                            Cramers V 0.29 Chi-squared   0.043
## c("sex", "lat")                          Cramers V 0.35 Chi-squared   0.044
## c("d", "csi")                           Pearsons r 0.35      F test   0.045
## c("sex", "sloc")                         Cramers V 0.44 Chi-squared   0.046
## c("fert", "ck18")                        Cramers V 0.28 Chi-squared   0.046
## c("csf", "os")                          Pearsons r 0.32      F test   0.046
## c("lm", "ck18")                          Cramers V 0.28 Chi-squared   0.046
## c("rt", "cd56")                          Cramers V 0.28 Chi-squared   0.046
## c("ct2", "ck17")                         Cramers V 0.28 Chi-squared   0.046
## c("p", "sma")                           Pearsons r 0.39      F test   0.046
## c("s100", "ck18")                        Cramers V 0.28 Chi-squared   0.046
## c("nfp", "nse")                          Cramers V 0.28 Chi-squared   0.046
## c("gfap", "ck18")                        Cramers V 0.28 Chi-squared   0.046
## c("ck", "ck1")                           Cramers V 0.28 Chi-squared   0.046
## c("ck", "ck2")                           Cramers V 0.28 Chi-squared   0.046
## c("ck", "ck3")                           Cramers V 0.28 Chi-squared   0.046
## c("ck", "ck4")                           Cramers V 0.28 Chi-squared   0.046
## c("ck", "ck5")                           Cramers V 0.28 Chi-squared   0.046
## c("ck", "ck6")                           Cramers V 0.28 Chi-squared   0.046
## c("ck", "ck10")                          Cramers V 0.28 Chi-squared   0.046
## c("ck", "ck14")                          Cramers V 0.28 Chi-squared   0.046
## c("ck", "ck15")                          Cramers V 0.28 Chi-squared   0.046
## c("ck", "ck16")                          Cramers V 0.28 Chi-squared   0.046
## c("ck8", "ck17")                         Cramers V 0.28 Chi-squared   0.046
## c("ck8", "ck18")                         Cramers V 0.28 Chi-squared   0.046
## c("ck17", "ck19")                        Cramers V 0.28 Chi-squared   0.046
## c("ck17", "ker")                         Cramers V 0.28 Chi-squared   0.046
## c("ck17", "midl")                        Cramers V 0.28 Chi-squared   0.046
## c("ck18", "ker")                         Cramers V 0.28 Chi-squared   0.046
## c("yr", "ice")                          Pearsons r 0.28      F test   0.047
## c("sex", "rt2")                          Cramers V 0.28 Chi-squared   0.047
## c("ct2", "ck10")                         Cramers V 0.28 Chi-squared   0.047
## c("ct2", "ck14")                         Cramers V 0.28 Chi-squared   0.047
## c("ct2", "ck15")                         Cramers V 0.28 Chi-squared   0.047
## c("ct2", "ck16")                         Cramers V 0.28 Chi-squared   0.047
```

```
## c("ini1", "nfp")                       Cramers V 0.28 Chi-squared   0.047
## c("synaptophysin", "chromograminA")  Cramers V 0.28 Chi-squared   0.047
## c("csf", "midl")                       Cramers V 0.28 Chi-squared   0.048
## c("yr", "nfp")                        Pearsons r 0.52      F test   0.049
## c("sloc", "itl")                       Cramers V 0.44 Chi-squared   0.049
## c("yr", "fert")                       Pearsons r 0.28      F test    0.05
## c("age", "midl")                      Pearsons r 0.28      F test   0.051
## c("drt", "ssloc")                      Cramers V 0.91 Chi-squared   0.051
## c("age", "lat")                       Pearsons r 0.35      F test   0.052
## c("sma", "src")                       Pearsons r 0.36      F test   0.052
## c("sex", "cd99")                       Cramers V 0.27 Chi-squared   0.053
## c("lm", "nfp")                         Cramers V 0.27 Chi-squared   0.053
## c("d", "tpd")                         Pearsons r 0.38      F test   0.053
## c("nfp", "gfap")                       Cramers V 0.27 Chi-squared   0.053
## c("rt2", "ema")                        Cramers V 0.27 Chi-squared   0.054
## c("ttp", "desmin")                    Pearsons r 0.47      F test   0.055
## c("lmr", "midl")                       Cramers V 0.27 Chi-squared   0.056
## c("sx", "midl")                        Cramers V 0.43 Chi-squared   0.058
## c("rt2", "ssloc")                      Cramers V 0.34 Chi-squared   0.058
## c("ck8", "ck10")                       Cramers V 0.27 Chi-squared   0.058
## c("ck8", "ck14")                       Cramers V 0.27 Chi-squared   0.058
## c("ck8", "ck15")                       Cramers V 0.27 Chi-squared   0.058
## c("ck8", "ck16")                       Cramers V 0.27 Chi-squared   0.058
## c("ck10", "ker")                       Cramers V 0.27 Chi-squared   0.058
## c("ck14", "ker")                       Cramers V 0.27 Chi-squared   0.058
## c("ck15", "ker")                       Cramers V 0.27 Chi-squared   0.058
## c("ck16", "ker")                       Cramers V 0.27 Chi-squared   0.058
## c("age", "cd99")                      Pearsons r 0.74      F test   0.059
## c("preg", "ker")                       Cramers V 0.27 Chi-squared    0.06
## c("drt", "sloc")                       Cramers V  1.2 Chi-squared    0.06
## c("lmr", "ttp")                       Pearsons r 0.38      F test   0.061
## c("sex", "sxb2")                       Cramers V 0.26 Chi-squared   0.062
## c("rt", "mib1n")                      Pearsons r 0.61      F test   0.062
## c("fert", "del22q")                    Cramers V 0.26 Chi-squared   0.063
## c("lm", "rt2")                         Cramers V 0.26 Chi-squared   0.063
## c("del22q", "gfap")                    Cramers V 0.26 Chi-squared   0.064
## c("s100", "cd34")                      Cramers V 0.26 Chi-squared   0.064
## c("lm", "os")                         Pearsons r 0.29      F test   0.065
## c("lm", "ker")                         Cramers V 0.26 Chi-squared   0.065
## c("gfap", "ck")                        Cramers V 0.26 Chi-squared   0.066
## c("ttp", "sxb2")                      Pearsons r 0.33      F test   0.067
```

9 Striking associations

```
options("xtable.include.rownames"=TRUE)
xtable(d1[, table(csf, gtr)])
```

	gtr	.
csf	FALSE	TRUE
no	0	3
yes	30	6

Table 39: csf × gtr
chi-sq=0.0099

```
xtable(d1[, table(src, sloc)])
```

	sloc	.	.	.	.
src	4v	lv	pin	pit	cord
0	3	14	0	6	4
1	3	3	5	4	0

Table 40: src × sloc
chi-sq=0.0065

```
xtable(d1[, table(lm, gfap)])
```

	gfap	.
lm	neg	pos
no	16	5
yes	2	7

Table 41: lm × gfap
chi-sq=0.018

```
xtable(d1[, table("age > 40"=age > 40,
                  del22q, useNA="ifany")])
```

	del22q	.	.
age > 40	neg	pos	NA
FALSE	0	10	25
TRUE	2	3	10

Table 42: age > 40 × del22q
chi-sq=0.082

```
xtable(d1[, table("age > 40"=age > 40, ssloc)])
```

	ssloc	.	.
age > 40	bs	lv	cord
FALSE	12	22	1
TRUE	6	4	5

Table 43: age > 40 × ssloc
chi-sq=0.0044

```
xtable(d1[, table(sma, synaptophysin)])
```

	synaptophysin	.
sma	neg	pos
neg	9	0
pos	5	6

Table 44: sma × synaptophysin
chi-sq=0.031

```
xtable(d1[, table(ema, ini1, useNA="ifany")])
```

	ini1	.	.
ema	neg	pos	NA
neg	2	1	3
pos	20	0	9
NA	1	0	14

Table 45: ema × ini1
chi-sq=0.00011

```
xtable(d1[, table(sex, midl)])
```

	midl	.
sex	FALSE	TRUE
m	15	7
f	9	17

Table 46: sex × midl
chi-sq=0.043

```
xtable(d1[, table(sex, sloc)])
```

	sloc	.	.	.	.
sex	4v	lv	pin	pit	cord
m	4	13	2	1	3
f	3	7	4	10	3

Table 47: sex × sloc
chi-sq=0.046

```
xtable(d1[, table(sex, nfp)])
```

	nfp	.
sex	neg	pos
m	5	4
f	0	6

Table 48: sex × nfp
chi-sq=0.094

```
xtable(d1[, table(s100, gfap)])
```

	gfap	.
s100	neg	pos
neg	9	2
pos	3	7

Table 49: s100 × gfap
chi-sq=0.051

```
xtable(d1[, table(lmr, ssloc)])
```

	ssloc	.	.
lmr	bs	lv	cord
no	2	16	2
yes	6	5	1

Table 50: lmr × ssloc
chi-sq=0.039

```
xtable(d1[, table(nfp, nse)])
```

	nse	.
nfp	neg	pos
neg	0	2
pos	2	0

Table 51: nfp × nse
chi-sq=0.32

```
xtable(d1[, table(nfp, ini1, useNA="ifany")])
```

	ini1	.	.
nfp	neg	pos	NA
neg	1	1	3
pos	7	0	3
NA	15	0	20

Table 52: nfp × ini1
chi-sq=0.016

```
xtable(d1[, table(chromograminA,
                  synaptophysin, useNA="ifany")])
```

	synaptophysin	.	.
chromograminA	neg	pos	NA
neg	7	1	1
pos	0	1	0
NA	9	4	27

Table 53: chromograminA × synaptophysin
chi-sq=0.00091

```
xtable(d1[, table(sex, cd99, useNA="ifany")])
```

	cd99	.	.
sex	neg	pos	NA
m	4	0	19
f	1	2	24

Table 54: sex × cd99
chi-sq=0.13

```
xtable(d1[, table(csf, midl, useNA="ifany")])
```

	midl	.	.
csf	FALSE	TRUE	NA
no	3	0	0
yes	16	23	1
NA	5	1	1

Table 55: csf × midl
chi-sq=0.059

```
xtable(d1[, table(lm, nfp, useNA="ifany")])
```

	nfp	.	.
lm	neg	pos	NA
no	5	5	25
yes	0	5	5
NA	0	0	5

Table 56: lm × nfp
chi-sq=0.051

```
xtable(d1[, table(gfap, nfp, useNA="ifany")])
```

	nfp	.	.
gfap	neg	pos	NA
neg	5	5	8
pos	0	5	7
NA	0	0	20

Table 57: gfap × nfp
chi-sq=0.00036

```
xtable(d1[, table(rt, ct, useNA="ifany")])
```

	ct	.	.
rt	no	yes	NA
no	8	1	0
yes	17	16	0
NA	0	0	8

Table 58: rt × ct
chi-sq=3.4e-11

```
xtable(d1[, table(rt2, ssloc, useNA="ifany")])
```

	ssloc	.	.
rt2	bs	lv	cord
no	2	10	1
yes	7	4	2
NA	9	12	3

Table 59: rt2 × ssloc
chi-sq=0.22

```
t1 <- d1[, table("age > 40"=age > 40, ssloc)]
xtable(t1)
```

	ssloc	.	.
age > 40	bs	lv	cord
FALSE	12	22	1
TRUE	6	4	5

Table 60: age > 40 × ssloc
chi-sq=0.0044

```
xtable(prop.table(t1, margin=2) * 100,
       digits=3,
       caption="Percentages (by colum)")
```

	ssloc	.	.
age > 40	bs	lv	cord
FALSE	66.7	84.6	16.7
TRUE	33.3	15.4	83.3

Table 61: Percentages (by colum)

```
d1[, assoc(age, ssloc)]
```

Pr(>F)1 "Pearsons r" "0.45" "F test" "0.0045"

```
d1[, assoc(age, ssloc=="cord")]
```

Pr(>F)1 "Pearsons r" "0.43" "F test" "0.0018"

```
xtable(d1[, table(sex, sloc, useNA="ifany")])
```

	sloc	.	.	.	.
sex	4v	lv	pin	pit	cord
m	4	13	2	1	3
f	3	7	4	10	3

Table 62: sex × sloc
chi-sq=0.046

```
xtable(d1[, table(sex, ssloc, useNA="ifany")])
```

	ssloc	.	.
sex	bs	lv	cord
m	5	15	3
f	13	11	3

Table 63: sex × ssloc
chi-sq=0.14

```
xtable(d1[, table(ssloc, lmr, useNA="ifany")])
```

	lmr	.	.
ssloc	no	yes	NA
bs	2	6	10
lv	16	5	5
cord	2	1	3

Table 64: ssloc × lmr
chi-sq=0.016

```
xtable(d1[, table(nestin, nfp, useNA="ifany")])
```

	nfp	.	.
nestin	neg	pos	NA
neg	0	0	0
pos	0	0	2
NA	5	10	33

Table 65: nestin × nfp
chi-sq=NaN

10 Time to progression (exploratory)

```
suppressWarnings(
  s1 <- sapply(d1, function(x) {
    tryCatch(summary(coxph(Surv(d1$ttp, d1$p) ~ x, data=d1))$sctest,
             error=function(e) rep(NaN, 3))}))
s1 <- as.data.frame(t(signif(s1, digits=2)))
s1 <- s1[order(s1$pvalue), ]
head(s1, 40)
```

```
##                 test df  pvalue
## mib1            82.0 17 1.6e-10
## first           99.0 30 2.6e-09
## ttp             21.0  1 5.1e-06
## d                9.9  1 1.6e-03
## notes           29.0 11 2.0e-03
## rt               8.1  1 4.4e-03
## p                8.1  1 4.4e-03
## sxb2             6.1  1 1.3e-02
## drt             25.0 13 2.0e-02
## sx              12.0  4 2.1e-02
## src              5.0  1 2.5e-02
## lmr              4.8  1 2.9e-02
## os               4.5  1 3.4e-02
## lm               4.2  1 4.1e-02
## ct               3.7  1 5.4e-02
## rt2              3.4  1 6.4e-02
## loc             19.0 12 7.9e-02
## ck18             2.9  1 9.0e-02
## csi              2.9  1 9.0e-02
## sx2              8.0  4 9.1e-02
## ema              2.5  1 1.2e-01
## synaptophysin    2.3  1 1.3e-01
## cd56             2.0  1 1.6e-01
## melanA           2.0  1 1.6e-01
## a1ACT            2.0  1 1.6e-01
## p53              2.0  1 1.6e-01
## mib1n            2.0  1 1.6e-01
## lat              3.6  2 1.7e-01
## desmin           1.9  1 1.7e-01
## sex              1.8  1 1.9e-01
## gfap             1.7  1 1.9e-01
## sma              1.7  1 2.0e-01
## chromograminA    1.6  1 2.1e-01
## csf              1.3  1 2.5e-01
## fert             1.2  1 2.7e-01
## del22q           1.2  1 2.8e-01
## it               2.4  2 2.9e-01
## midl             1.1  1 3.0e-01
## age              1.0  1 3.1e-01
## olig2            1.0  1 3.2e-01
```

11 Time to progression

```
s2 <- suppressWarnings(
  sapply(d1[, list(rt, sx, gfap, lm, midl,
                   sloc, ck18, ct, synaptophysin,
                   ema, a1ACT, melanA, p53,
                   cd56, csf, sex, csi, gtr)],
         function(x){
           c1 <- coxph(Surv(d1$ttp, d1$p) ~ x, data=d1)
           c("n="=c1$n,
             exp(coef(c1)),
             summary(c1)$sctest["pvalue"])}))
options("xtable.include.rownames"=TRUE)
for (i in seq(length(s2))) {
  print(names(s2[i]))
  print(xtable(
    d1[, table(get(names(s2[i])), p)]))
  print(xtable(
    as.data.frame(s2[[i]]),
    caption=names(s2[i])))
}
```

[1] "rt"

	p	.
	0	1
no	0	8
yes	4	23

Table 66: × p
chi-sq=0.6

	s2[[i]]
n=	33.00
xyes	0.31
pvalue	0.00

Table 67: rt

[1] "sx"

	p	.
	0	1
none	0	1
bx	0	2
str	2	15
sx	2	6
gtr	0	7

Table 68: × p
chi-sq=0.6

	s2[[i]]
n=	33.00
x.L	0.30
x.Q	0.76
x.C	4.46
x^4	0.56
pvalue	0.02

Table 69: sx

[1] "gfap"

	p	.
	0	1
neg	2	12
pos	1	7

Table 70: × p
chi-sq=1

	s2[[i]]
n=	22.00
xpos	1.91
pvalue	0.19

Table 71: gfap

[1] "lm"

	p	.
	0	1
no	3	24
yes	1	7

Table 72: × p
chi-sq=1

	s2[[i]]
n=	33.00
xyes	2.43
pvalue	0.04

Table 73: lm

[1] "midl"

	p	.
	0	1
FALSE	3	15
TRUE	1	15

Table 74: × p
chi-sq=0.68

	s2[[i]]
n=	32.00
xTRUE	1.51
pvalue	0.30

Table 75: midl

[1] "sloc"

	p	.
	0	1
4v	2	4
lv	1	13
pin	0	4
pit	1	7
cord	0	3

Table 76: × p
chi-sq=0.4

	s2[[i]]
n=	33.00
xlv	1.18
xpin	1.26
xpit	1.85
xcord	2.43
pvalue	0.71

Table 77: sloc

[1] "ck18"

	p	.
	0	1
neg	0	2
pos	0	2

Table 78: × p
chi-sq=NaN

	s2[[i]]
n=	4.00
xpos	2598857513.66
pvalue	0.09

Table 79: ck18

[1] "ct"

	p	.
	0	1
no	0	23
yes	4	8

Table 80: × p
chi-sq=0.017

	s2[[i]]
n=	33.00
xyes	0.44
pvalue	0.05

Table 81: ct

[1] "synaptophysin"

	p	.
	0	1
neg	1	11
pos	2	3

Table 82: × p
chi-sq=0.39

	s2[[i]]
n=	15.00
xpos	0.35
pvalue	0.13

Table 83: synaptophysin

[1] "ema"

	p	.
	0	1
neg	0	4
pos	4	21

Table 84: × p
chi-sq=0.94

	s2[[i]]
n=	27.00
xpos	0.42
pvalue	0.12

Table 85: ema

[1] "a1ACT"

	p	.
	0	1
neg	0	1
pos	0	2

Table 86: × p
chi-sq=NaN

	s2[[i]]
n=	3.00
xpos	0.00
pvalue	0.16

Table 87: a1ACT

[1] "melanA"

	p	.
	0	1
neg	0	4
pos	0	1

Table 88: × p
chi-sq=NaN

	s2[[i]]
n=	5.00
xpos	0.00
pvalue	0.16

Table 89: melanA

[1] "p53"

	p	.
	0	1
neg	0	1
pos	0	4

Table 90: × p
chi-sq=NaN

	s2[[i]]
n=	5.00
xpos	1048969603.23
pvalue	0.16

Table 91: p53

[1] "cd56"

	p	.
	0	1
neg	0	2
pos	0	1

Table 92: × p
chi-sq=NaN

	s2[[i]]
n=	3.00
xpos	4170531059.85
pvalue	0.16

Table 93: cd56

[1] "csf"

	p	.
	0	1
no	0	2
yes	3	27

Table 94: × p
chi-sq=1

	s2[[i]]
n=	30.00
xyes	3.15
pvalue	0.25

Table 95: csf

[1] "sex"

	p	.
	0	1
m	4	13
f	0	18

Table 96: × p
chi-sq=0.098

	s2[[i]]
n=	33.00
xf	1.66
pvalue	0.19

Table 97: sex

[1] "csi"

	p	.
	0	1
FALSE	1	24
TRUE	2	1

Table 98: × p
chi-sq=0.02

	s2[[i]]
n=	28.00
xTRUE	0.21
pvalue	0.09

Table 99: csi

[1] "gtr"

	p	.
	0	1
FALSE	4	24
TRUE	0	7

Table 100: × p
chi-sq=0.69

	s2[[i]]
n=	33.00
xTRUE	0.86
pvalue	0.75

Table 101: gtr

```
cat("\\textbf{\\Large{Multivariable model}} \\newline")
```

Multivariable model

```
xtable(coxph(Surv(ttp, d) ~ rt + gtr + gfap + lm, data=d1))
```

	coef	exp(coef)	se(coef)	z	p
rtyes	-1.15	0.32	0.75	-1.53	0.13
gtrTRUE	-0.88	0.41	0.69	-1.28	0.20
gfappos	0.82	2.26	0.61	1.34	0.18
lmyes	1.49	4.44	0.61	2.43	0.01

12 Overall survival (exploratory)

```
suppressWarnings(
  s1 <- sapply(d1, function(x) {
    tryCatch(summary(coxph(Surv(d1$os, d1$d) ~ x, data=d1))$sctest,
             error=function(e) rep(NaN, 3))}))
s1 <- as.data.frame(t(signif(s1, 2)))
s1 <- s1[order(s1$pvalue), ]
head(s1, 20)
```

```
##        test df  pvalue
## first 130.0 36 6.4e-12
## d      25.0  1 6.7e-07
## mib1   58.0 18 4.1e-06
## notes  45.0 13 2.0e-05
## os     13.0  1 3.3e-04
## lm     13.0  1 3.5e-04
## ttp    12.0  1 6.2e-04
## drt    36.0 14 1.2e-03
## sx     17.0  4 2.0e-03
## mib1n   6.6  1 1.0e-02
## it     10.0  3 1.6e-02
## src     5.8  1 1.6e-02
## tpd     4.7  1 3.1e-02
## csf     4.4  1 3.6e-02
## gfap    4.3  1 3.8e-02
## p       4.1  1 4.2e-02
## preg    3.8  1 5.1e-02
## ct      3.5  1 6.3e-02
## lmr     3.4  1 6.7e-02
## csi     2.9  1 8.7e-02
```

13 Overall survival

```
s2 <- suppressWarnings(
  sapply(d1[, list(lm, sx, gfap, src,
                   csf, preg, csi, lmr,
                   ct, melanA, ice, gtr)],
         function(x) {
           c1 <- coxph(Surv(d1$os, d1$d) ~ x,
                       data=d1)
           c("n="=c1$n,
             exp(coef(c1)),
             summary(c1)$sctest["pvalue"])}))
for (i in seq(length(s2))) {
  print(names(s2[i]))
  print(xtable(
    d1[, table(get(names(s2[i])), d)]))
  print(xtable(
    as.data.frame(s2[[i]]),
    caption=names(s2[i])))
}
```

[1] "lm"

	d	.
	0	1
no	13	18
yes	2	8

Table 102: × d
chi-sq=0.38

	s2[[i]]
n=	41.00
xyes	4.59
pvalue	0.00

Table 103: lm

[1] "sx"

	d	.
	0	1
none	0	1
bx	0	3
str	8	11
sx	3	5
gtr	5	4

Table 104: × d
chi-sq=0.46

	s2[[i]]
n=	40.00
x.L	0.16
x.Q	0.91
x.C	2.71
x^4	0.43
pvalue	0.00

Table 105: sx

[1] "gfap"

	d	.
	0	1
neg	8	8
pos	4	7

Table 106: × d
chi-sq=0.76

	s2[[i]]
n=	27.00
xpos	3.09
pvalue	0.04

Table 107: gfap

[1] "src"

	d	.
	0	1
0	4	21
1	12	3

Table 108: × d
chi-sq=0.00025

	s2[[i]]
n=	40.00
x	0.25
pvalue	0.02

Table 109: src

[1] "csf"

	d	.
	0	1
no	3	0
yes	10	27

Table 110: × d
chi-sq=0.051

	s2[[i]]
n=	40.00
xyes	262345330.72
pvalue	0.04

Table 111: csf

[1] "preg"

	d	.
	0	1
no	9	7
yes	0	2

Table 112: × d
chi-sq=0.45

	s2[[i]]
n=	18.00
xyes	4.49
pvalue	0.05

Table 113: preg

[1] "csi"

	d	.
	0	1
FALSE	9	19
TRUE	4	1

Table 114: × d
chi-sq=0.13

	s2[[i]]
n=	33.00
xTRUE	0.20
pvalue	0.09

Table 115: csi

[1] "lmr"

	d	.
	0	1
no	7	11
yes	1	9

Table 116: × d
chi-sq=0.24

	s2[[i]]
n=	28.00
xyes	2.35
pvalue	0.07

Table 117: lmr

[1] "ct"

	d	.
	0	1
no	4	19
yes	12	5

Table 118: × d
chi-sq=0.0022

	s2[[i]]
n=	40.00
xyes	0.39
pvalue	0.06

Table 119: ct

[1] "melanA"

	d	.
	0	1
neg	0	4
pos	0	1

Table 120: × d
chi-sq=NaN

	s2[[i]]
n=	5.00
xpos	0.00
pvalue	0.16

Table 121: melanA

[1] "ice"

	d	.
	0	1
FALSE	11	29
TRUE	5	1

Table 122: × d
chi-sq=0.027

	s2[[i]]
n=	46.00
xTRUE	0.30
pvalue	0.21

Table 123: ice

[1] "gtr"

	d	.
	0	1
FALSE	11	20
TRUE	5	4

Table 124: × d
chi-sq=0.49

	s2[[i]]
n=	40.00
xTRUE	0.58
pvalue	0.33

Table 125: gtr

```
cat("\\textbf{\\Large{Multivariable models}} \\newline")
```

Multivariable models

```
d2 <- d1[, list(lm, gfap,
                sx, ct, rt,
                os, d)]
xtable(coxph(Surv(os, d) ~ ., data=d2))
```

	coef	exp(coef)	se(coef)	z	p
lmyes	2.47	11.78	0.96	2.57	0.01
gfappos	0.56	1.74	1.02	0.54	0.59
sx.L	-2.98	0.05	1.20	-2.49	0.01
sx.Q	0.19	1.21	1.08	0.18	0.86
sx.C	-0.74	0.48	1.32	-0.56	0.58
sx^4	-1.38	0.25	0.85	-1.63	0.10
ctyes	-1.84	0.16	0.79	-2.34	0.02
rtyes	0.55	1.73	0.85	0.65	0.52

```
xtable(coxph(Surv(os, d) ~ gtr, data=d1))
```

	coef	exp(coef)	se(coef)	z	p
gtrTRUE	-0.54	0.58	0.57	-0.96	0.34

```
d2 <- d1[, list(lm, gfap,
                gtr, ct, rt,
                os, d)]
xtable(coxph(Surv(os, d) ~ ., data=d2))
```

	coef	exp(coef)	se(coef)	z	p
lmyes	1.71	5.50	0.75	2.29	0.02
gfappos	0.89	2.43	0.77	1.16	0.25
gtrTRUE	-1.28	0.28	0.72	-1.78	0.07
ctyes	-1.43	0.24	0.67	-2.12	0.03
rtyes	0.41	1.51	0.79	0.52	0.60

```
d2 <- d1[, list(lm, gfap,
                src,
                os, d)]
xtable(coxph(Surv(os, d) ~ ., data=d2))
```

	coef	exp(coef)	se(coef)	z	p
lmyes	1.79	6.00	0.91	1.97	0.05
gfappos	0.59	1.80	0.97	0.61	0.54
src	-1.82	0.16	0.79	-2.30	0.02

```
setkey(d1, lm)
cat("\\onecolumn")
```

```
autoplot(ten(Surv(os, d) ~ lm, data=d1[os < 36, ]),
         title="Time to death by leptomeningeal metastases at diagnosis",
         timeTicks="months",
         survLineSize=3,
         legTitle="LM", legLabs=c("no", "yes"),
         legTitleSize=18, legLabSize=15, legorder=c(1, 0))
```

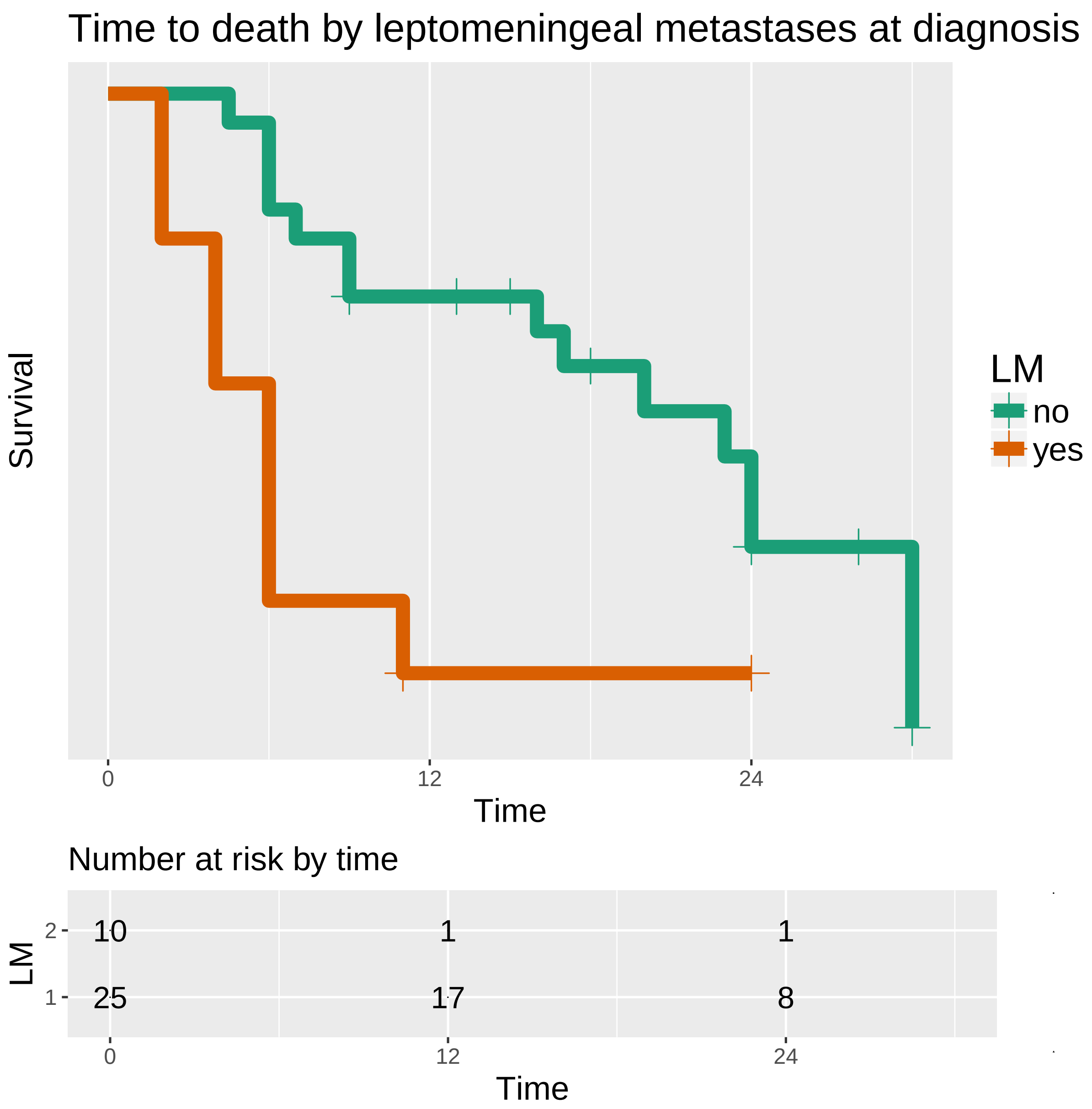

14 Progression to death (exploratory)

```
suppressWarnings(
  s1 <- sapply(d1, function(x){
    tryCatch(summary(coxph(Surv(d1$tpd, d1$d) ~ x, data=d1))$sctest,
             error=function(e) rep(NaN, 3))}))
s1 <- as.data.frame(t(signif(s1, 2)))
s1 <- s1[order(s1$pvalue), ]
head(s1, 20)
```

```
##       test df  pvalue
## first 80.0 25 1.3e-07
## drt   42.0  9 3.1e-06
## lm    13.0  1 3.0e-04
## mib1  33.0 13 1.5e-03
## os    10.0  1 1.6e-03
## notes 30.0 11 1.6e-03
## gfap   9.2  1 2.4e-03
## tpd    8.1  1 4.4e-03
## d      7.1  1 7.8e-03
## actin  5.1  1 2.5e-02
## it     4.5  1 3.5e-02
## itl    4.5  1 3.5e-02
## mib1n  3.9  1 4.8e-02
## ttp    3.4  1 6.4e-02
## ck18   2.9  1 9.0e-02
## csf    2.3  1 1.3e-01
## dct    8.4  5 1.3e-01
## ker    2.1  1 1.5e-01
## sxb    1.9  1 1.6e-01
## ct     1.8  1 1.8e-01
```

15 Progression to death

```
suppressWarnings(
  s2 <- sapply(d1[, list(lm, gfap, actin,
                         csf, ker, sxb,
                         csi, ct, preg)],
               function(x) {
                 c1 <- coxph(Surv(d1$tpd, d1$d) ~ x,
                             data=d1)
                 c(" n="=c1$n,
                   exp(coef(c1)),
                   summary(c1)$sctest["pvalue"])},
             simplify=FALSE))
for (i in seq(length(s2))) {
  print(names(s2[i]))
  print(xtable(
    d1[, table(get(names(s2[i])), d)]))
  print(xtable(
    as.data.frame(s2[[i]]),
    caption=names(s2[i])))
}
```

[1] "lm"

	d	.
	0	1
no	13	18
yes	2	8

Table 126: × d
chi-sq=0.38

	s2[[i]]
n=	27.00
xyes	6.71
pvalue	0.00

Table 127: lm

[1] "gfap"

	d	.
	0	1
neg	8	8
pos	4	7

Table 128: × d
chi-sq=0.76

	s2[[i]]
n=	18.00
xpos	7.49
pvalue	0.00

Table 129: gfap

[1] "actin"

	d	.
	0	1
neg	1	2
pos	1	3

Table 130: × d
chi-sq=1

	s2[[i]]
n=	7.00
xpos	3381371571.90
pvalue	0.02

Table 131: actin

[1] "csf"

	d	.
	0	1
no	3	0
yes	10	27

Table 132: × d
chi-sq=0.051

	s2[[i]]
n=	25.00
xyes	223923092.25
pvalue	0.13

Table 133: csf

[1] "ker"

	d	.
	0	1
neg	8	20
pos	8	10

Table 134: × d
chi-sq=0.43

	s2[[i]]
n=	27.00
xpos	1.92
pvalue	0.15

Table 135: ker

[1] "sxb"

	d	.
	0	1
FALSE	0	1
TRUE	16	23

Table 136: × d
chi-sq=1

	s2[[i]]
n=	27.00
xTRUE	0.25
pvalue	0.16

Table 137: sxb

[1] "csi"

	d	.
	0	1
FALSE	9	19
TRUE	4	1

Table 138: × d
chi-sq=0.13

	s2[[i]]
n=	23.00
xTRUE	0.35
pvalue	0.30

Table 139: csi

[1] "ct"

	d	.
	0	1
no	4	19
yes	12	5

Table 140: × d
chi-sq=0.0022

	s2[[i]]
n=	27.00
xyes	2.04
pvalue	0.18

Table 141: ct

[1] "preg"

	d	.
	0	1
no	9	7
yes	0	2

Table 142: × d
chi-sq=0.45

	s2[[i]]
n=	11.00
xyes	2.94
pvalue	0.19

Table 143: preg

```
xtable(survfit(Surv(tpd, d) ~ lm, data=d1))
```

	records	n.max	n.start	events	median	0.95LCL	0.95UCL
lm=no	20	20	20	15	15	8	50
lm=yes	7	7	7	7	5	1	NA

Table 144: Survival for Surv(tpd, d) lm

```
xtable(survfit(Surv(tpd, d) ~ gfap, data=d1))
```

	records	n.max	n.start	events	median	0.95LCL	0.95UCL
gfap=neg	12	12	12	8	13	8	NA
gfap=pos	6	6	6	6	4.5	1	NA

Table 145: Survival for Surv(tpd, d) gfap

```
cat("\\textbf{\\Large{Multivariable model}} \\newline")
```

Multivariable model

```
xtable(coxph(Surv(tpd, d) ~ lm + gfap, data=d1))
```

	coef	exp(coef)	se(coef)	z	p
lmyes	1.31	3.71	1.48	0.89	0.37
gfappos	0.86	2.37	1.48	0.59	0.56

16 Recursive partitioning

```
suppressPackageStartupMessages(library("rpart"))
d2 <- d1[, list("LM  "=lm, "GFAP  "=gfap,
                " Sx  "=sx, gtr, rt, ct, "SRC  "=src,
                preg, os, d)]
r1 <- rpart(Surv(os, d) ~ ., data=d2, method="exp")
plot(r1, uniform=TRUE, compress=TRUE, margin=0.05)
text(r1, all=TRUE, use.n=TRUE, pretty=TRUE,
     fancy=TRUE, fheight=3, digits=2)
d2 <- d2[, 'LM  ' := NULL]
r1 <- rpart(Surv(os, d) ~ ., data=d2, method="exp")
plot(r1, uniform=TRUE, margin=0.1)
text(r1, all=TRUE, use.n=TRUE, pretty=TRUE, fancy=TRUE, digits=2)
```

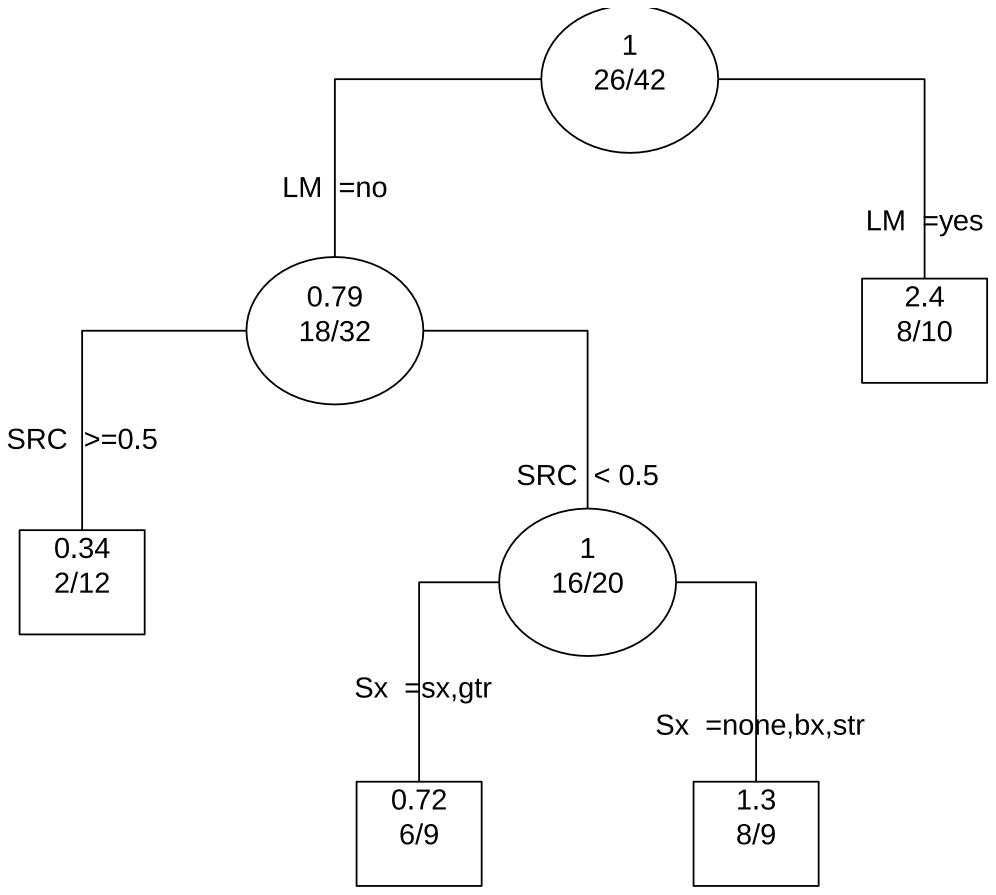

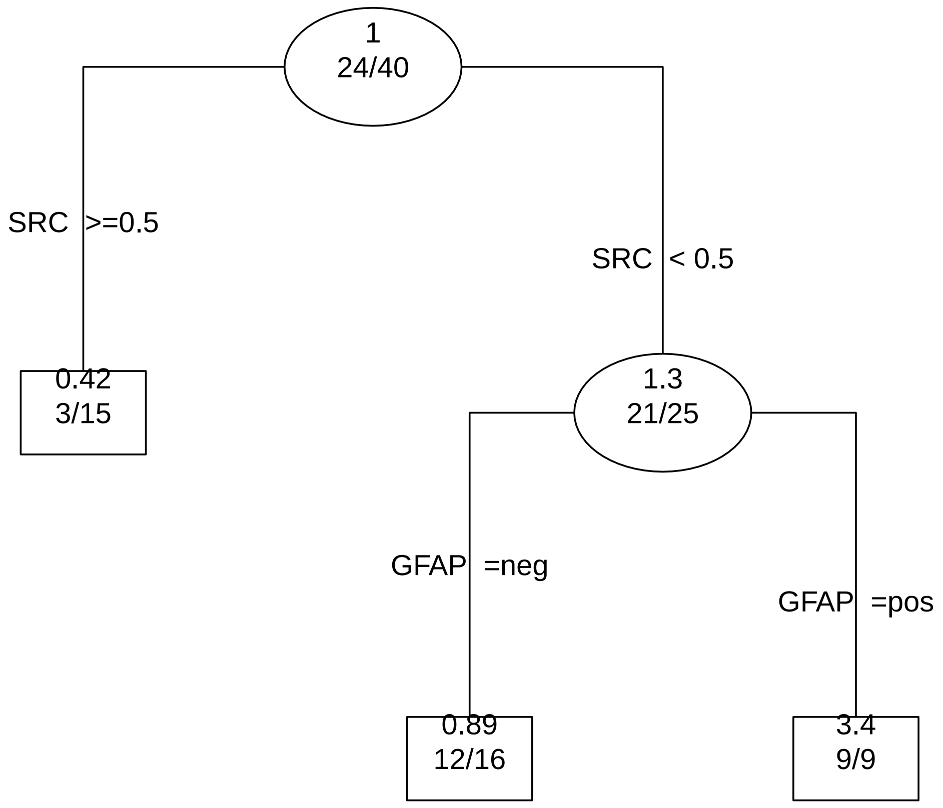
1
24/40
SRC >=0.5
SRC < 0.5
0.42
3/15
1.3
21/25
GFAP =neg
GFAP =pos
0.89
12/16
3.4
9/9

17 Multiple imputation

```
suppressPackageStartupMessages(library("mice"))
d2 <- d1[, list(lm, gfap,
                sx, gtr, rt, ct, src,
                preg)]
d2 <- d2[, src := as.logical(src)]
set.seed(1)
suppressWarnings(
    c1 <- capture.output({
        m1 <- mice(d2, m=10)
    }))
c1 <- complete(m1)
c2 <- cbind(c1, d1[, list(os, d)])
c2 <- c2[complete.cases(c2), ]
l1 <- length(c2)
s2 <- suppressWarnings(
  sapply(c2[, -((l1 - 1):l1)],
         function(x){
           c1 <- coxph(Surv(c2$os, c2$d) ~ x, data=c2)
           c("n="=c1$n,
             exp(coef(c1)),
             summary(c1)$sctest["pvalue"])}))
options("xtable.include.rownames"=TRUE)
for (i in seq(length(s2))) {
  print(xtable(
    as.data.frame(s2[[i]]),
    caption=names(s2[i])))
}
```

	s2[[i]]
n=	46.00
x2	3.57
pvalue	0.00

Table 146: lm

	s2[[i]]
n=	46.00
x2	1.57
pvalue	0.24

Table 147: gfap

	s2[[i]]
n=	46.00
x2	1.07
x3	0.32
x4	0.26
x5	0.27
pvalue	0.11

Table 148: sx

	s2[[i]]
n=	46.00
x	0.89
pvalue	0.79

Table 149: gtr

	s2[[i]]
n=	46.00
x2	0.72
pvalue	0.47

Table 150: rt

	s2[[i]]
n=	46.00
x2	0.52
pvalue	0.12

Table 151: ct

	s2[[i]]
n=	46.00
x	0.41
pvalue	0.04

Table 152: src

	s2[[i]]
n=	46.00
x2	0.96
pvalue	0.93

Table 153: preg

```
r1 <- with(c2, rpart(Surv(os, d) ~ ., data=c2))
plot(r1, uniform=TRUE, margin=0.1)
text(r1, all=TRUE, use.n=TRUE, pretty=TRUE, fancy=TRUE, digits=2)
```

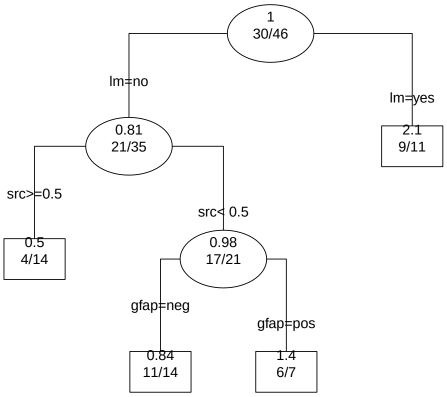

18 Using Nested cohort to contol

```
suppressPackageStartupMessages(library("NestedCohort"))
cat("\\textbf{\\Large{lm}} \\newline")
```

lm

```
c1 <- nested.coxph("Surv(os, d) ~ sx + rt + ct",
                   data=d1[!(is.na(os)|is.na(lm)), ],
                   samplingmod="lm*d")
xtable(c1)
```

	coef	exp(coef)	se(coef)	z	p
sx.L	-2.09	0.12	0.68	-3.06	0.00
sx.Q	0.23	1.26	0.48	0.48	0.63
sx.C	0.30	1.34	0.54	0.55	0.58
sx^4	-0.54	0.58	0.35	-1.53	0.12
rtyes	-0.37	0.69	0.61	-0.60	0.55
ctyes	-0.84	0.43	0.55	-1.53	0.13

```
c1 <- nested.coxph("Surv(os, d) ~ gtr + rt + ct",
                   data=d1[!(is.na(os)|is.na(lm)), ],
                   samplingmod="lm*d")
xtable(c1)
```

	coef	exp(coef)	se(coef)	z	p
gtrTRUE	-0.85	0.43	0.83	-1.02	0.31
rtyes	-0.58	0.56	0.55	-1.05	0.30
ctyes	-0.81	0.45	0.47	-1.72	0.09

```
c1 <- nested.coxph("Surv(os, d) ~ src",
                   data=d1[!(is.na(os)|is.na(lm)), ],
                   samplingmod="lm*d")
xtable(c1)
```

	coef	exp(coef)	se(coef)	z	p
src	-1.30	0.27	0.59	-2.19	0.03

```
cat("\\textbf{\\Large{gfap}} \\newline")
```

gfap

```
c1 <- nested.coxph("Surv(os, d) ~ sx + rt + ct",
                   data=d1[!(is.na(os)|is.na(gfap)), ],
                   samplingmod="gfap*d")
xtable(c1)
```

	coef	exp(coef)	se(coef)	z	p
sx.L	-2.58	0.08	0.49	-5.24	0.00
sx.Q	-0.18	0.83	0.47	-0.39	0.70
sx.C	0.51	1.66	0.93	0.54	0.59
sx^4	-1.19	0.30	0.53	-2.25	0.02
rtyes	-0.20	0.82	0.79	-0.26	0.80
ctyes	-1.35	0.26	0.39	-3.49	0.00

```
c1 <- nested.coxph("Surv(os, d) ~ gtr + rt + ct",
                   data=d1[!(is.na(os)|is.na(gfap)), ],
                   samplingmod="gfap*d")
xtable(c1)
```

	coef	exp(coef)	se(coef)	z	p
gtrTRUE	-1.18	0.31	0.76	-1.56	0.12
rtyes	-0.44	0.64	0.65	-0.68	0.50
ctyes	-1.16	0.31	0.38	-3.08	0.00

```
c1 <- nested.coxph("Surv(os, d) ~ src",
                   data=d1[!(is.na(os)|is.na(gfap)), ],
                   samplingmod="gfap*d")
xtable(c1)
```

	coef	exp(coef)	se(coef)	z	p
src	-1.57	0.21	0.61	-2.59	0.01

19 Session Info

```
toLatex(sessionInfo())
```

- R version 3.3.1 (2016-06-21), x86_64-pc-linux-gnu
- Locale: LC_CTYPE=en_US.UTF-8, LC_NUMERIC=C, LC_TIME=en_US.UTF-8, LC_COLLATE=en_US.UTF-8, LC_MONETARY=en_US.UTF-8, LC_MESSAGES=en_US.UTF-8, LC_PAPER=en_US.UTF-8, LC_NAME=C, LC_ADDRESS=C, LC_TELEPHONE=C, LC_MEASUREMENT=en_US.UTF-8, LC_IDENTIFICATION=C
- Base packages: base, graphics, grDevices, methods, splines, stats, utils
- Other packages: car 2.0-20, data.table 1.9.6, heplots 1.0-12, knitr 1.12.3, lattice 0.20-29, MASS 7.3-33, mice 2.22, NestedCohort 1.1-3, Rcpp 0.12.7, rpart 4.1-8, survival 2.37-7, survMisc 0.5.4, xtable 1.7-3

- Loaded via a namespace (and not attached): assertthat 0.1, chron 2.3-45, colorspace 1.2-4, digest 0.6.4, evaluate 0.8.3, formatR 0.10, ggplot2 2.2.1, grid 3.3.1, gridExtra 0.9.1, gtable 0.1.2, highr 0.6, km.ci 0.5-2, KMsurv 0.1-5, labeling 0.2, lazyeval 0.2.0, munsell 0.4.2, nnet 7.3-8, plyr 1.8.1, randomForest 4.6-10, RColorBrewer 1.0-5, scales 0.4.1, stringr 0.6.2, tibble 1.2, tools 3.3.1, zoo 1.7-11

References

[1] A. J. Balaton, P. Vaury, and M. Videgrain. Paravertebral malignant rhabdoid tumor in an adult. A case report with immunocytochemical study. *Pathol. Res. Pract.*, 182(5):713–718, Oct 1987. doi:10.1016/S0344-0338(89)80130-X. [PubMed:2446296].

[2] M. Horn, W. Schlote, K. D. Lerch, W. I. Steudel, D. Harms, and E. Thomas. Malignant rhabdoid tumor: primary intracranial manifestation in an adult. *Acta Neuropathol.*, 83(4):445–448, 1992. doi:10.1007/BF00713540. [PubMed:1575023].

[3] A. Cossu, G. Massarelli, V. Manetto, G. Viale, F. Tanda, L. Bosincu, P. Iuzzolino, S. Cossu, R. Padovani, and V. Eusebi. Rhabdoid tumours of the central nervous system. Report of three cases with immunocytochemical and ultrastructural findings. *Virchows Arch A Pathol Anat Histopathol*, 422(1):81–85, 1993. doi:10.1007/BF01605137. [PubMed:7679853].

[4] B. J. Fisher, J. Siddiqui, D. Macdonald, A. E. Cairney, D. Ramsey, D. Munoz, and R. Del Maestro. Malignant rhabdoid tumor of brain: an aggressive clinical entity. *Can J Neurol Sci*, 23(4):257–263, Nov 1996. doi:10.1017/S0317167100038191. [PubMed:8951203].

[5] R. Ashraf, R. C. Bentley, A. N. Awan, R. E. McLendon, and M. W. Ragozzino. Implantation metastasis of primary malignant rhabdoid tumor of the brain in an adult (one case report). *Med. Pediatr. Oncol.*, 28(3):223–227, Mar 1997. doi:10.1002/(SICI)1096-911X(199703)28:3<223::AID-MPO14>3.0.CO;2-F. [PubMed:9024522].

[6] D. Byram. Regarding Weiss et al., IJROBP 41:103-109; 1998. *Int. J. Radiat. Oncol. Biol. Phys.*, 45(1): 247, Aug 1999. doi:10.1017/CBO9781107415324.004. [PubMed:10477033].

[7] Y. Sugita, Y. Takahashi, I. Hayashi, M. Morimatsu, K. Okamoto, and M. Shigemori. Pineal malignant rhabdoid tumor with chondroid formation in an adult. *Pathol. Int.*, 49(12):1114–1118, Dec 1999. doi:10.1046/j.1440-1827.1999.00988.x. [PubMed:10632935].

[8] A. Kuge, T. Kayama, D. Tsuchiya, K. Kawakami, S. Saito, Y. Nakazato, and H. Suzuki. [Suprasellar primary malignant rhabdoid tumor in an adult: a case report]. *No Shinkei Geka*, 28(4):351–358, Apr 2000. [PubMed:10769834].

[9] J. Arrazola, I. Pedrosa, R. Mendez, C. Saldana, B. W. Scheithauer, and A. Martinez. Primary malignant rhabdoid tumour of the brain in an adult. *Neuroradiology*, 42(5):363–367, May 2000. doi:10.1016/j.jocn.2009.02.011. [PubMed:10872158].

[10] J. Lutterbach, J. Liegibel, D. Koch, A. Madlinger, H. Frommhold, and A. Pagenstecher. Atypical teratoid/rhabdoid tumors in adult patients: case report and review of the literature. *J. Neurooncol.*, 52(1):49–56, Mar 2001. doi:10.1023/A:1010683416555. [PubMed:11451202].

[11] L. A. Bruch, D. A. Hill, D. X. Cai, B. K. Levy, L. P. Dehner, and A. Perry. A role for fluorescence in situ hybridization detection of chromosome 22q dosage in distinguishing atypical teratoid/rhabdoid tumors from medulloblastoma/central primitive neuroectodermal tumors. *Hum. Pathol.*, 32(2):156–162, Feb 2001. doi:10.1053/hupa.2001.21572. [PubMed:11230702].

[12] J. Pimentel, R. Silva, and T. Pimentel. Primary malignant rhabdoid tumors of the central nervous system: considerations about two cases of adulthood presentation. *J. Neurooncol.*, 61(2):121–126, Jan 2003. doi:10.1023/A:1022135518846. [PubMed:12622450].

[13] R. Kachhara, T. M. Retnam, S. Kumar, S. Nair, R. N. Bhattacharya, T. Krishnamoorthy, and V. V. Radhakrishnan. Rhabdoid tumor of the thalamus. *Neurol India*, 51(2):273–274, Jun 2003. [PubMed:14571026].

[14] T. Kawaguchi, T. Kumabe, M. Watanabe, and T. Tominaga. Atypical teratoid/rhabdoid tumour with leptomeningeal dissemination in an adult. *Acta Neurochir (Wien)*, 146(9):1033–1038, Sep 2004. doi:10.1007/s00701-004-0313-5. [PubMed:15340816].

[15] J. Raisanen, J. A. Biegel, K. J. Hatanpaa, A. Judkins, C. L. White, and A. Perry. Chromosome 22q deletions in atypical teratoid/rhabdoid tumors in adults. *Brain Pathol.*, 15(1):23–28, Jan 2005. doi:10.1111/j.1750-3639.2005.tb00096.x. [PubMed:15779233].

[16] M. L. Erickson, R. Johnson, S. I. Bannykh, A. de Lotbiniere, and J. H. Kim. Malignant rhabdoid tumor in a pregnant adult female: literature review of central nervous system rhabdoid tumors. *J. Neurooncol.*, 74(3):311–319, Sep 2005. doi:10.1007/s11060-004-7560-4. [DOI:10.1007/s11060-004-7560-4] [PubMed:16132523].

[17] Y. W. Chen, T. T. Wong, D. M. Ho, P. I. Huang, K. P. Chang, C. Y. Shiau, and S. H. Yen. Impact of radiotherapy for pediatric CNS atypical teratoid/rhabdoid tumor (single institute experience). *Int. J. Radiat. Oncol. Biol. Phys.*, 64(4):1038–1043, Mar 2006. doi:10.1016/j.ijrobp.2005.10.001. [PubMed:16406394].

[18] B. Ingold, M. Moschopulos, G. Hutter, H. Seeger, B. Rothlisberger, H. Landolt, Y. Yonekawa, W. Jochum, and F. L. Heppner. Abdominal seeding of an atypical teratoid/rhabdoid tumor of the pineal gland along a ventriculoperitoneal shunt catheter. *Acta Neuropathol.*, 111(1):56–59, Jan 2006. doi:10.1007/s00401-005-1112-7. [PubMed:16328512].

[19] T. Rezanko, M. Tunakan, A. Kahraman, H. K. Sucu, F. Gelal, and I. Akkol. Primary rhabdoid tumor of the brain in an adult. *Neuropathology*, 26(1):57–61, Feb 2006. doi:10.1111/j.1440-1789.2006.00624.x. [PubMed:16521480].

[20] G. Chacko, A. G. Chacko, C. P. Dunham, A. R. Judkins, J. A. Biegel, and A. Perry. Atypical teratoid/rhabdoid tumor arising in the setting of a pleomorphic xanthoastrocytoma. *J. Neurooncol.*, 84(2):217–222, Sep 2007. doi:10.1007/s11060-007-9361-z. [PubMed:17431546].

[21] E. L. Zarovnaya, H. F. Pallatroni, E. B. Hug, P. A. Ball, L. D. Cromwell, J. M. Pipas, C. E. Fadul, L. P. Meyer, J. P. Park, J. A. Biegel, A. Perry, and C. H. Rhodes. Atypical teratoid/rhabdoid tumor of the spine in an adult: case report and review of the literature. *J. Neurooncol.*, 84(1):49–55, Aug 2007. doi:10.1007/s11060-007-9339-x. [PubMed:17377740].

[22] A. T. Makuria, E. J. Rushing, K. M. McGrail, D. P. Hartmann, N. Azumi, and M. Ozdemirli. Atypical teratoid rhabdoid tumor (AT/RT) in adults: review of four cases. *J. Neurooncol.*, 88(3): 321–330, Jul 2008. doi:10.1007/s11060-008-9571-z. [PubMed:18369529].

[23] K. Arita, K. Sugiyama, T. Sano, and H. Oka. Atypical teratoid/rhabdoid tumour in sella turcica in an adult. *Acta Neurochir (Wien)*, 150(5):491–495, May 2008. doi:10.1007/s00701-008-1500-y. [PubMed:18309453].

[24] V. Samaras, A. Stamatelli, E. Samaras, I. Stergiou, P. Konstantopoulou, V. Varsos, A. R. Judkins, J. A. Biegel, and C. Barbatis. Atypical teratoid/rhabdoid tumor of the central nervous system in an 18-year-old patient. *Clin. Neuropathol.*, 28(1):1–10, 2009. [PubMed Central:PMC2712356] [PubMed:19216214].

[25] H. Takei, A. M. Adesina, V. Mehta, S. Z. Powell, and L. A. Langford. Atypical teratoid/rhabdoid tumor of the pineal region in an adult. *J. Neurosurg.*, 113(2):374–379, Aug 2010. doi:10.3171/2009.10.JNS09964. [PubMed:19911885].

[26] N. A. Shonka, T. S. Armstrong, S. S. Prabhu, A. Childress, S. Choi, L. A. Langford, and M. R. Gilbert. Atypical teratoid/rhabdoid tumors in adults: a case report and treatment-focused review. *J Clin Med Res*, 3(2):85–92, Apr 2011. doi:10.4021/jocmr535w. [PubMed Central:PMC3140928][PubMed:21811535].

[27] K. Takahashi, H. Nishihara, M. Katoh, T. Yoshinaga, R. Mahabir, H. Kanno, T. Kimura, M. Tanino, J. Ikeda, Y. Sawamura, K. Nagashima, and S. Tanaka. Case of atypical teratoid/rhabdoid tumor in an adult, with long survival. *Brain Tumor Pathol*, 28(1):71–76, Feb 2011. doi:10.1007/s10014-010-0008-y. [PubMed:21181449].

[28] P. Gorayski, S. Boros, B. Ong, S. Olson, and M. Foote. Radiation-induced primary cerebral atypical teratoid/rhabdoid tumour in an adult. *J Clin Neurosci*, 20(10):1466–1468, Oct 2013. doi:10.1016/j.jocn.2013.03.041. [PubMed:24018258].

[29] C. Moretti, D. Lupoi, F. Spasaro, L. Chioma, P. Di Giacinto, M. Colicchia, M. Frajoli, R. Mocini, S. Ulisse, M. Antonelli, F. Giangaspero, and L. Gnessi. Sella turcica atypical teratoid/rhabdoid tumor complicated with lung metastasis in an adult female. *Clin Med Insights Case Rep*, 6:177–182, 2013. doi:10.4137/CCRep.S12834. [PubMed Central:PMC3855097] [PubMed:24324353].

[30] S. Roy, C. Mallik, S. Maiti, and T. Chaudhuri. Temporal lobe atypical teratoid/ rhabdoid tumor in a 24-year old adult female. *South Asian J Cancer*, 2(4):210, Oct 2013. doi:10.4103/2278-330X.119909. [PubMed Central:PMC3889036] [PubMed:24455633].

[31] H. G. Park, J. H. Yoon, S. H. Kim, K. H. Cho, H. J. Park, S. H. Kim, and E. H. Kim. Adult-onset sellar and suprasellar atypical teratoid rhabdoid tumor treated with a multimodal approach: a case report. *Brain Tumor Res Treat*, 2(2):108–113, Oct 2014. doi:10.14791/btrt.2014.2.2.108. [PubMed Central:PMC4231618] [PubMed:25408935].

[32] S. Shitara and Y. Akiyama. Atypical teratoid/rhabdoid tumor in sellar turcica in an adult: A case report and review of the literature. *Surg Neurol Int*, 5:75, 2014. doi:10.4103/2152-7806.133105. [PubMed Central:PMC4061576] [PubMed:24949218].

[33] C. Souki, M. Abdel-Rhaman, A. Qasem, and M. Al-Hussaini. Atypical teratoid rhabdoid tumor in adulthood. *Clin. Neuropathol.*, 33(3):245–250, 2014. doi:10.5414/NP300683. [PubMed:24569171].

[34] X. Wang, X. Liu, Z. Lin, Y. Chen, P. Wang, and S. Zhang. Atypical teratoid/rhabdoid tumor (AT/RT) arising from the acoustic nerve in a young adult: a case report and a review of literature. *Medicine (Baltimore)*, 94(4):e439, Jan 2015. doi:10.1097/MD.0000000000000439. [PubMed Central:PMC4602954] [PubMed:25634176].

[35] M. Kanoto, Y. Toyoguchi, T. Hosoya, M. Kuchiki, and Y. Sugai. Radiological image features of the atypical teratoid/rhabdoid tumor in adults: a systematic review. *Clin Neuroradiol*, 25(1):55–60, Mar 2015. doi:10.1007/s00062-013-0282-2. [PubMed:24477665].

[36] S. Liebigt, A. Florschutz, N. Arndt, K. Stock, and C. Renner. Atypical Teratoid/Rhabdoid Tumor of the Pineal Region in a Young Adult Male Patient: Case Report and Review of the Literature. *J Neurol Surg A Cent Eur Neurosurg*, pages 1–7, May 2016. doi:10.1055/s-0036-1583180. [PubMed:27144539].

[37] JM Regan, M Tehrani, FJ Rodriguez, and JC Watson. Adult At/Rt: Predilection for the Pituitary? *J. Neurol. Neurosurg.*, 2(1):110, 2015. doi:10.19104/jnn.2015.110.

[38] P. Sinha, M. Ahmad, A. Varghese, T. Parekh, A. Ismail, A. Chakrabarty, A. Tyagi, and P. Chumas. Atypical teratoid rhabdoid tumour of the spine: report of a case and literature review. *Eur Spine J*, 24 Suppl 4:S472–484, May 2015. doi:10.1007/s00586-014-3445-1. [PubMed:25374299].

[39] ZS Dreyfuss and T Simpson. A Pediatric Tumor Causing A Neurosurgical Emergency In An Adult. In *Am J Respir Crit Care Med*, volume 193, page A1965, 2016.

[40] Christopher Dardis, Eric C Woolf, and Adrienne C Scheck. Towards reproducible research: From data analysis (in r) to a typeset laboratory notebook (as. pdf) using the text editor emacs with the'mp'package. *F1000Research*, 4, 2015. doi:10.12688/f1000research.6800.2.
